# Modulation of functional pendant chains within poly(ethylene glycol) hydrogels for refined control of protein release

**DOI:** 10.1038/s41598-018-22249-1

**Published:** 2018-03-12

**Authors:** Mirae Kim, Chaenyung Cha

**Affiliations:** School of Materials Science and Engineering, Ulsan National Institute of Science and Technology, Ulsan, 44919 South Korea

## Abstract

Hydrogels are highly attractive delivery vehicles for therapeutic proteins. Their innate biocompatibility, hydrophilicity and aqueous permeability allow stable encapsulation and release of proteins. The release rates also can be controlled simply by altering the crosslinking density of the polymeric network. However, the crosslinking density also influences the mechanical properties of hydrogels, generally opposite to the permeability. In addition, the release of larger proteins may be hindered below critically diminished porosity determined by the crosslinking density. Herein, the physical properties of the hydrogels are tuned by presenting functional pendant chains, independent of crosslinking density. Heterobifunctional poly(ethylene glycol) monomethacrylate (PEGMA) with various end functional groups is synthesized and copolymerized with PEG dimethacrylate (PEGDA) to engineer PEG hydrogels with pendant PEG chains. The pendant chains of the PEG hydrogels consisting of sulfonate, trimethylammonium chloride, and phenyl groups are utilized to provide negative charge, positive charge and hydrophobicity, respectively, to the hydrogels. The release rates of proteins with different isoelectric points are controlled in a wide range by the type and the density of functional pendant chains via electrostatic and hydrophobic interactions.

## Introduction

Hydrogels are a popular class of carriers for various biological molecules, such as proteins and DNA, as well as cells and tissues for biomedical applications^1–4^. Highly favorable physical properties of hydrogels prepared using biocompatible polymers, such as elasticity and hydrophilicity, help provide the encapsulated entities with optimal mechanical and aqueous environment and protection for prolonged life-time. In addition, the fabrication methods are generally mild enough to allow for safe encapsulation with high efficiency. The cellular activities within the hydrogels could be further promoted by modifying the polymer network with cell-responsive moieties^5,6^.

For controlled release applications, the release rates of encapsulated biomolecules from hydrogels are often controlled in an efficient manner by simply varying the crosslinking density of the polymeric network, which in effect controls the porosity of the hydrogel^7–9^. However, this approach is also often encountered with a few critical drawbacks. First, the crosslinking density, according to the rubber elasticity theory, also affects the mechanical properties of the hydrogels, in which the mechanical and diffusional properties are inversely correlated^10,11^. Thus, this interdependency could lead to an inadvertent change in mechanical properties while attempting to control the release rates via crosslinking density; hydrogels may become structurally too weak at lower crosslinking density to increase the release rate, or they may become too brittle at higher crosslinking density. Furthermore, the release may be severely hindered for the hydrogel having significantly reduced pores, which is especially critical for the release of larger macromolecules such as large proteins and DNA^8,12^. To overcome these issues, it would be ideal to control the release rates of encapsulated species in a wide range while minimally affecting the crosslinking density of the hydrogels.

**Figure 1:**
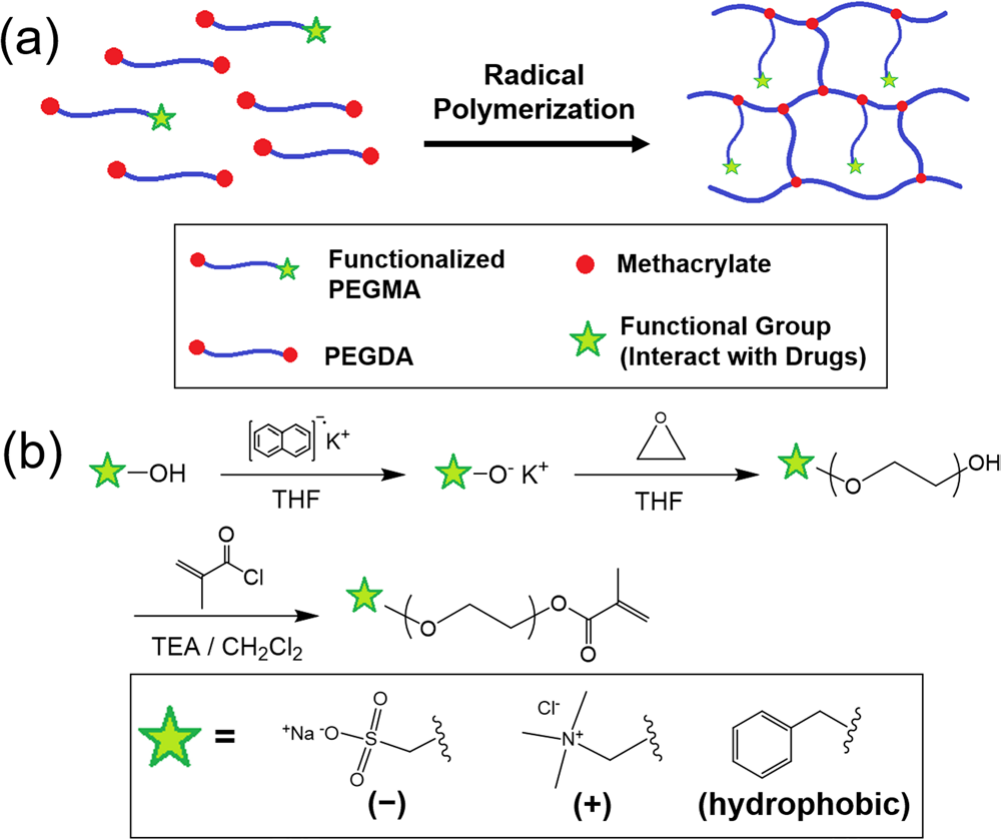
(**a**) Fabrication of PEG hydrogels with functional pendant chains via radical copolymerization of PEGDA with heterobifunctional PEGMA. (**b**) Synthesis of heterobifunctional PEGMA via anionic polymerization of epoxide with an initiator containing the desired functional group, followed by the conjugation of methacrylate. The functional groups used in this study were sulfonate (negative charge), trimethylammonium chloride (positive charge) and phenyl (hydrophobicity) groups.

In this study, protein release rates from hydrogels were controlled by varying the physical properties of the hydrogels while maintaining their crosslinking density. Poly(ethylene glycol) dimethacrylate (PEGDA) hydrogels presenting pendant chains with end functional groups with varying physical properties (i.e. charge density and hydrophobicity) were fabricated by radical copolymerization with heterobifunctional PEG monomethacrylate (PEGMA) (Fig. 1a). There have been previously published reports of engineering charged or hydrophobic hydrogels, but their approaches generally involved utilizing the monomers or macromers with charged or hydrophobic functional domains (e.g. acrylate, vinyl sulfonate, [2-(methacryloxy)ethyl] trimethylammonium chloride), poloxamers, and heparin), and thus maintaining their crosslinking densities were not factored^13–19^. However, in this study, by changing the number of “functional” pendant chains while keeping the total number of pendant chains constant, the crosslinking density of the PEG hydrogels could be controlled, thereby minimally affecting the mechanical properties, while varying the physical properties of the hydrogels. With PEG pendant chains with charged functional groups, (e.g. negatively charged sulfonate and positively charged trimethylammonium chloride), the release of proteins having different charge densities at physiological pH due to different isoelectric points (pI) could be controlled via electrostatic interaction. Three model proteins, namely albumin, insulin and trypsin, having different pI values, were used. Their release rates from the PEG hydrogels with varying number of charged pendant chains were measured. Furthermore, since proteins have varying degrees of hydrophobicity based on amino acid compositions, the release from PEG hydrogels with hydrophobic phenyl pendant chains was also explored.

## Results and Discussion

### Synthesis of heterobifunctional PEGMA

PEG hydrogels have long been used as drug delivery systems due to biocompatibility, abundant source, bioinertness, and efficient processing^6,20^. In particular, PEG with vinyl end groups, such as PEGDA, are widely used to develop hydrogels via radical polymerization either by itself or hybridizing with other vinyl-based polymers. The drug release rates from PEG hydrogels can be accomplished by controlling the crosslinking density which in turn controls the porosity of the polymeric network^7,8,21^. The physical properties of the PEG hydrogels can be further varied by introducing different moieties into the PEG network; copolymerization with other polymers or hybridizing with nanostructures (i.e. nanocomposite hydrogels)^22,23^. However, these strategies are often accompanied by unwanted changes in mechanical and swelling properties of the hydrogels, which may complicate the control of drug release.

Here, PEG hydrogels presented with varying number of pendant PEG chains with functional end groups were developed by copolymerizing PEGDA with heterobifunctional PEG monomethacrylate (PEGMA) to control the physical properties (e.g. charge density and hydrophobicity) while maintaining the crosslinking density (Fig. 1a). By keeping the number of pendant chains, it would prevent unwanted change in mechanical and swelling properties that could also influence the release of encapsulated drugs.

Three different types of heterobifunctional PEGMA was synthesized; sodium sulfoethyl PEGMA (Sulfo-PEGMA), trimethylammonium chloride PEGMA (TMAC-PEGMA), and phenyl PEGMA (Ph-PEGMA) in order to impart negative charge, positive charge and hydrophobicity, respectively (Fig. 1b). The ring-opening polymerization of epoxide with the anionic initiator having the desired functional group was employed to synthesize the heterobifunctional PEG, which was followed by the conjugation of methacrylate via nucleophilic substitution of the hydroxyl group of PEG using methacryloyl chloride. The chemical compositions of various heterobifunctional PEGMA were confirmed with ^1^H-NMR and ^13^C-NMR (see Supplementary Figs S1 and S2). The zeta potentials of Sulfo-PEGMA and TMAC-PEGMA were also determined to be −0.208 mV and 0.11 mV (0.1 wt% in 10 mM NaCl), respectively, which further confirmed the difference in the type and density of charges (see Supplementary Fig. S3).

### Mechanical properties of PEG hydrogel with functional pendant chains

**Figure 2:**
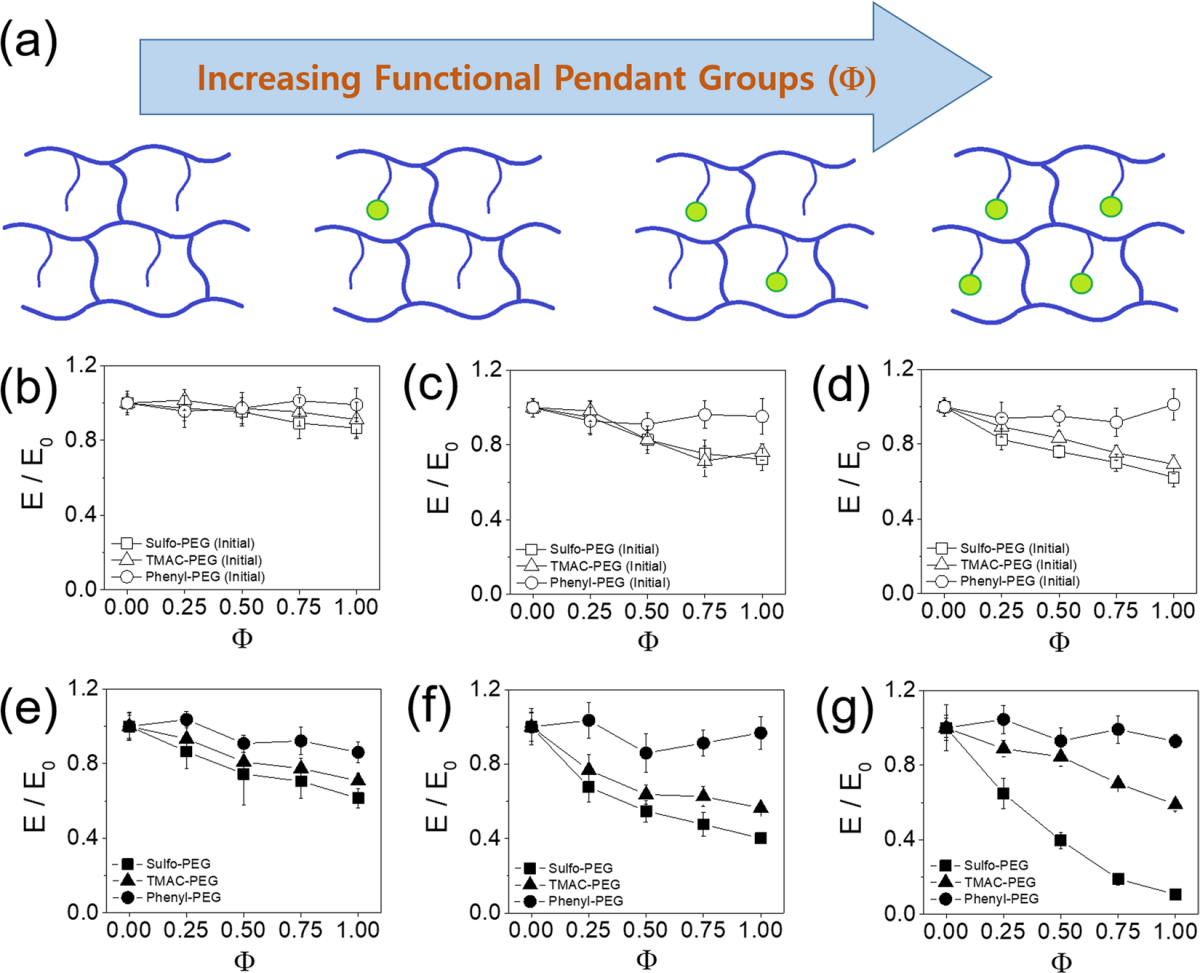
(**a**) The fraction of functional pendant chains (Φ) was varied while keeping the total number of pendant chains constant. Normalized elastic moduli (E/E_0_) of PEG hydrogels with varying Φ, measured (**b**,**c**,**d**) right after fabrication and (**e**,**f**,**g**) after incubation in PBS for 24 hours. The moduli were normalized with that at Φ = 0 (E_0_). (**b**,**e**) 10% PEGDA and 2% PEGMA, (**c**,**f**) 8% PEGDA and 4% PEGMA, (**d**,**g**) 6% PEGDA and 6% PEGMA.

In order to control the physical properties of PEG hydrogels while minimizing the difference in crosslinking density among different pendant chains, the total number of pendant chains were kept constant while varying the fraction of functional pendant chains (Φ) (Fig. 2a). This was accomplished by varying the ratio of heterobifunctional PEGMA with methoxy PEGMA as a non-functional control, while keeping the total concentration of PEGMA in a precursor solution (e.g. Φ = 0 when all the pendant chains are non-functional, Φ = 1 when all the pendant chains are functional).

The rigidity of the PEG hydrogels with varying Φ was measured to assess the effect of functional pendant chains. The concentrations of PEGDA and PEGMA were first kept at 10% and 2% (w/v) respectively. Increasing Φ did not result in a significant change in elastic moduli of the hydrogels, and the moduli at a given Φ were similar for all types of pendant chains (Fig. 2b, see Supplementary Fig. S4). This result demonstrated that the crosslinking density of hydrogels at varying Φ was not significantly affected for all types of pendant chains at the given PEGDA and PEGMA concentrations.

**Figure 3:**
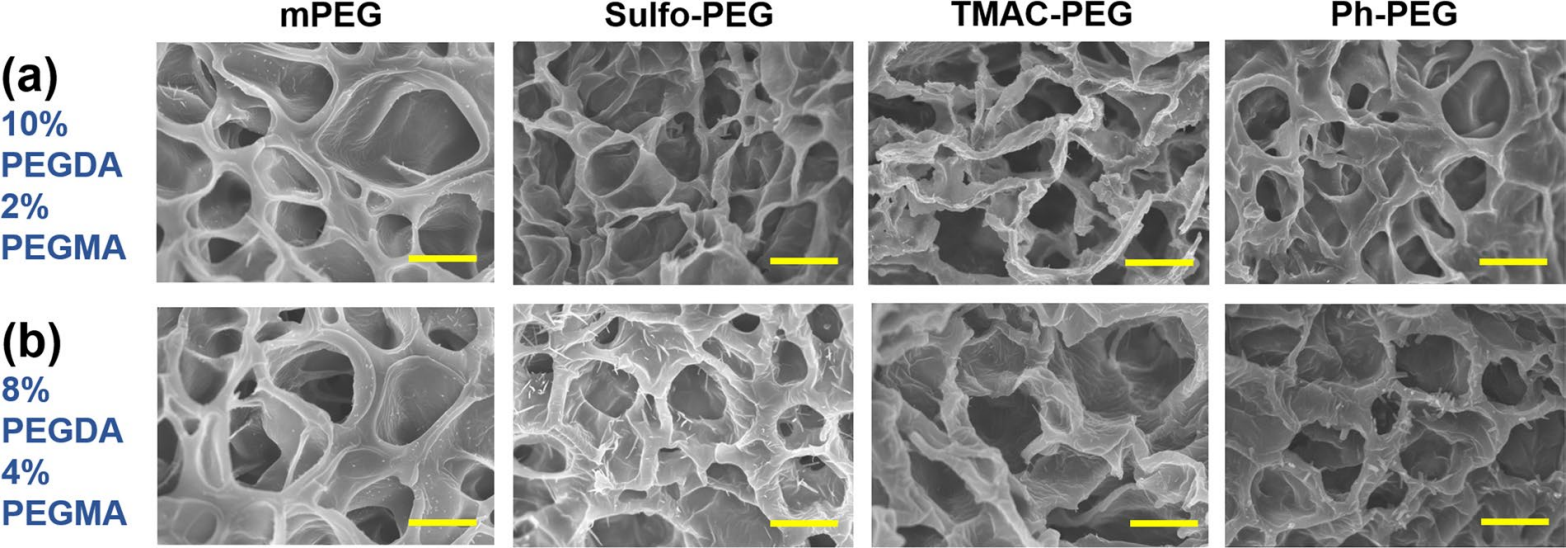
Scanning electron microscopic (SEM) images of non-functional mPEG hydrogel, Sulfo-PEG hydrogel, TMAC-PEG hydrogel, and Ph-PEG hydrogel at Φ = 1 (scale bar: 5 μm). (**a**) 10% PEGDA and 2% PEGMA, (**b**) 8% PEGDA and 4% PEGMA.

The relative concentrations of PEGDA and PEGMA were varied to further evaluate the possible change in crosslinking density by varying Φ; the PEGMA concentration was increased to 4% and 6% (w/v) while decreasing PEGDA concentration to 8% and 6% (w/v), thus keeping the total polymer concentration at 12% (w/v). Since the PEGDA molecules are mainly responsible for the crosslinking of the polymeric network, the overall elastic moduli became smaller at higher PEGMA concentrations (see Supplementary Fig. S4). There was a small decrease in moduli above Φ = 0.5 for Sulfo-PEG and TMAC-PEG hydrogels, and the decrease became larger at higher PEGMA concentration (e.g. 15% and 25% decreases at Φ = 1 for 4% and 6% PEGMA, respectively) (Fig. 2c,d, see Supplementary Fig. S5). This result may have been due to the increased charge density pushing the polymer chains further apart via repulsion, leading to a diminished degree of crosslinking. It is further corroborated by the lack of change in moduli of uncharged Ph-PEG hydrogels regardless of Φ and PEGMA concentration. Scanning electron microscopic (SEM) images of the hydrogels with 2% and 4% PEGMA also showed that the porosity, which generally correlates with the crosslinking density, was not significantly affected by the type of pendant chains at a given Φ (Fig. 3)^24,25^. Taken together, except at the higher Φ of Sulfo-PEG and TMAC-PEG hydrogels with lower crosslinking density (i.e. higher PEGMA concentration), the mechanical properties of the hydrogels were generally well maintained at varying Φ. Even in those conditions, the crosslinking densities of Sulfo-PEG and TMAC-PEG hydrogels at a given Φ were similar, and thus the protein release from the two hydrogel systems could be analyzed based only on the difference in charge density.

Next, the moduli of the hydrogels were measured after incubation in PBS in order to evaluate the effect of swelling into hydrogels controlled by different charge densities (Fig. 2e–g, see Supplementary Figs S5 and S6). It was hypothesized that the decrease in moduli by the swelling would be varied by the type and density of charge within the hydrogels. The moduli of Ph-PEG hydrogels were not affected by Φ, as expected, regardless of the concentrations of PEGDA and PEGMA, due to the lack of charge. However, the decrease in moduli of Sulfo-PEG hydrogels by Φ became more prominent with decreasing PEGDA (increasing PEGMA), especially more so than TMAC-PEG hydrogels. For example, the moduli of Sulfo-PEG hydrogels decreased with Φ by 60% and 85% for hydrogels with 8% PEGDA and 4% PEGMA and 6% PEGDA and 6% PEGMA, respectively. On the other hand, the moduli of TMAC-PEG hydrogels decreased with Φ only by 40% and 50% for hydrogels with 8% PEGDA and 4% PEGMA and 6% PEGDA and 6% PEGMA, respectively. These results indicated that (1) the increased charge density within the Sulfo-PEG hydrogels and TMAC-PEG hydrogels with increasing Φ likely pushed the polymer chains further apart via repulsion during swelling and resulted in greater chain relaxation, and (2) the larger decrease in moduli for Sulfo-PEG hydrogels compared to TMAC-hydrogels is likely due to the greater charge density of the Sulfo group compared to TMAC groups, as evidenced by their zeta potentials^26,27^.

**Figure 4:**
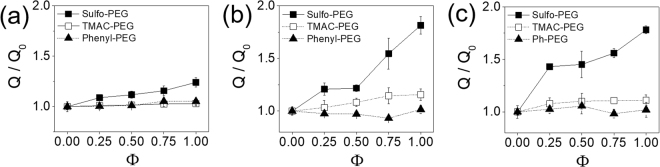
Normalized swelling ratios (Q/Q_0_) of PEG hydrogels with varying Φ. The swelling ratios were normalized with that at Φ = 0 (Q_0_). (**a**) 10% PEGDA and 2% PEGMA, (**b**) 8% PEGDA and 4% PEGMA, (**c**) 6% PEGDA and 6% PEGMA.

The trends of swelling ratios of PEG hydrogels with functional pendant chains were in conjunction with those of elastic moduli shown in Fig. 2, as expected, since the degree of swelling and rigidity are inversely related according to the rubber elasticity theory (Fig. 4). Interestingly, the swelling ratios of TMAC-PEG hydrogels were not significantly affected by Φ especially compared to the decrease in moduli. It is likely a result of smaller ionic strength and increased local hydrophobicity by the methyl groups in TMAC, preventing further swelling behavior. This is further highlighted in comparison with the Sulfo-PEG hydrogels, which do not have hydrophobic groups and have greater ionic strength than TMAC, showing much greater increase in swelling ratios with Φ.

### Protein release from PEG hydrogel with functional pendant chains

Three proteins having different isoelectric points (pI), albumin, insulin, and trypsin, were used as model protein drugs, and their release profiles from the PEG hydrogels with varying Φ were measured. Albumin and insulin have the pI values of 4.9 and 5.3, so they are expected to be negatively charged at the physiological pH of 7.4. The pI of trypsin, on the other hand, is 10.5, thus it would be positively charged at the physiological pH. By comparing their release profiles, the effect of charge type and density in the hydrogels could be evaluated. Furthermore, between albumin and insulin, their molecular weights are 66 kDa and 5.8 kDa, respectively, so the effect of the size of the protein on the release could also be determined. The protein release profiles with respect to time was then fitted with either (1) the Ritger-Peppas model in order to obtain the kinetic rate constants (*k*) and exponents (*n*) (Eq. (1)), or the Fickian diffusion model to obtain diffusion coefficients (*D*) (Eq. (2)), in order to analyze the drug release kinetics. Compared to the traditional Fickian diffusion model which is based on a fixed square root dependence on time, the Ritger-Peppas model allows the determination of both kinetic rate constant and power-law dependence on time^28^. The exponents indicate the release mechanism which depends heavily on the permeability and physical characteristics (e.g. chain relaxation) of the drug carriers. For hydrogels which undergo significant swelling due to the osmotic pressure from the surrounding medium as well as the chain relaxation of the polymeric network, the Fickian diffusion model is often limited when describing the overall release profiles (i.e. this model is more accurate at the initial protein release in which the release is mostly governed by the diffusion, whereas the release by chain relaxation deviates from the square root time dependence).

#### Albumin

**Figure 5:**
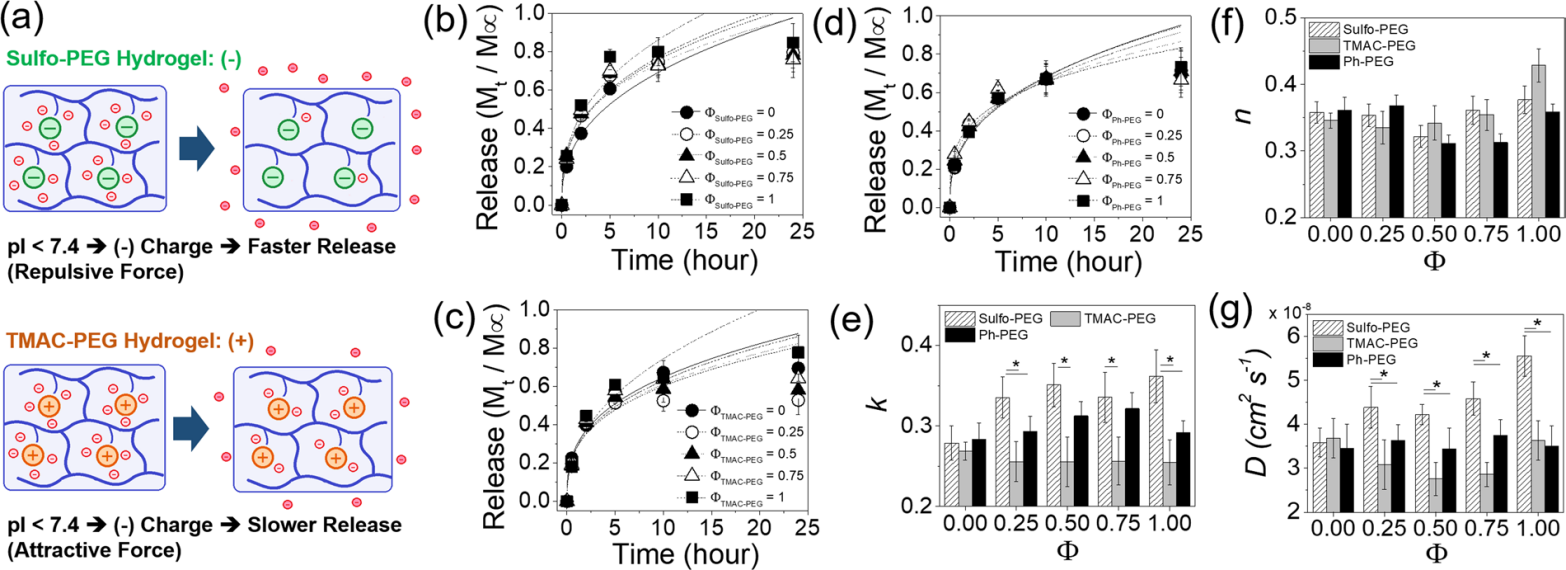
(**a**) Protein with pI lower than 7.4 becomes negatively charged, and thus expected to have a greater release rate in Sulfo-PEG hydrogels via repulsion than TMAC-PEG hydrogels. The release profiles of albumin from (**b**) Sulfo-PEG hydrogels, (**c**) TMAC-PEG hydrogels and (**d**) Ph-PEG hydrogels. (**e**) The kinetic rate constants (*k*) and (**f**) exponents (*n*) were obtained by fitting the curves with Eq. (1). (**g**) Diffusion coefficients (*D*) were obtained by fitting the curves with Eq. (2). The PEGDA and PEGMA concentrations of the hydrogels were 8% and 4%, respectively. (*p < 0.05).

Albumin is the most abundant type of protein commonly found in the plasma of most mammalian species, responsible for the transport of various molecules and maintaining the oncotic pressure^29^. It is negatively charged at physiological pH due to its lower pI value of 4.9. Therefore, the albumin encapsulated in Sulfo-PEG hydrogel was expected to have higher release rates than that of TMAC-PEG hydrogels due to electrostatic repulsion (Fig. 5a). The albumin release profiles from Sulfo-PEG hydrogels and TMAC-PEG hydrogels were measured (Fig. 5b,c), and the kinetic rate constants (*k*) were obtained via Ritger-Peppas model (Fig. 5e). The concentrations of PEGDA and PEGMA were first kept at 8% and 4%, respectively. As expected, the *k* values of Sulfo-PEG hydrogels increased with Φ, and were significantly larger than those of TMAC-PEG hydrogels at all Φ, demonstrating that the increased negative charge by Sulfo-PEG facilitated the release of albumin (Fig. 5e). Although the *k* values in TMAC-PEG hydrogels were lower than those of Sulfo-PEG hydrogels, increasing Φ did not result in the decrease of *k* values via electrostatic attraction as expected, likely due to the lower charge density of TMAC-PEG compared to Sulfo-PEG (i.e. TMAC-PEG has the lower absolute value of zeta potential than Sulfo-PEG).

The release profiles and their *k* values of albumin from PEG hydrogels with phenyl groups (Ph-PEG) were also obtained not only as a control to the Sulfo-PEG and TMAC-PEG hydrogels but also as a hydrogel with hydrophobicity (Fig. 5d and e). The *k* values of Ph-PEG hydrogels at each Φ was in between those of Sulfo-PEG and TMAC-PEG hydrogel, demonstrating their charge neutrality. The values were not as significantly influenced by the increase in Φ, which showed the increase in hydrophobicity had little effect on the interaction between albumin and the polymeric network.

The exponents (*n*) from the Ritger-Peppas models were between 0.3 and 0.4 for all conditions, indicating the drug release was governed mostly by the Fickian diffusion and the release mechanism was not affected by the type of pendant functional groups (Fig. 5f)^30–32^. However, the release mechanism here showed smaller time-dependency (e.g. greater initial release) than a pure Fickian diffusion (i.e. the *n* value for a pure Fickian diffusion is 0.5). This ‘quasi’ Fickian diffusion mechanism suggested that the presence of more freely moving pendant chains, as compared to fully linked chains with limited mobility, likely promoted the faster drug release than what would be expected for the pure Fickian diffusion.

The drug release profiles were alternatively fitted with a Fickian diffusion model (Eq. (2)) which assumes the square root of time dependence (~*t*^1/2^), and obtained the diffusion coefficients (*D*) (Fig. 5g, see Supplementary Fig. S7). Because of the greater time dependence, the Fickian diffusion model did not fit well after first 10 hours of release (i.e. approximately 60% of total release) and overestimated the *D* values compared to *k* values from the Ritger-Peppas model^33^. Nonetheless, the trend in *D* values were similar to that of *k* values shown in Fig. 5e, as they were expected to correlate with each other if *n* values from the Ritger-Peppas model were close to 0.5 and similar among different conditions.

**Figure 6:**
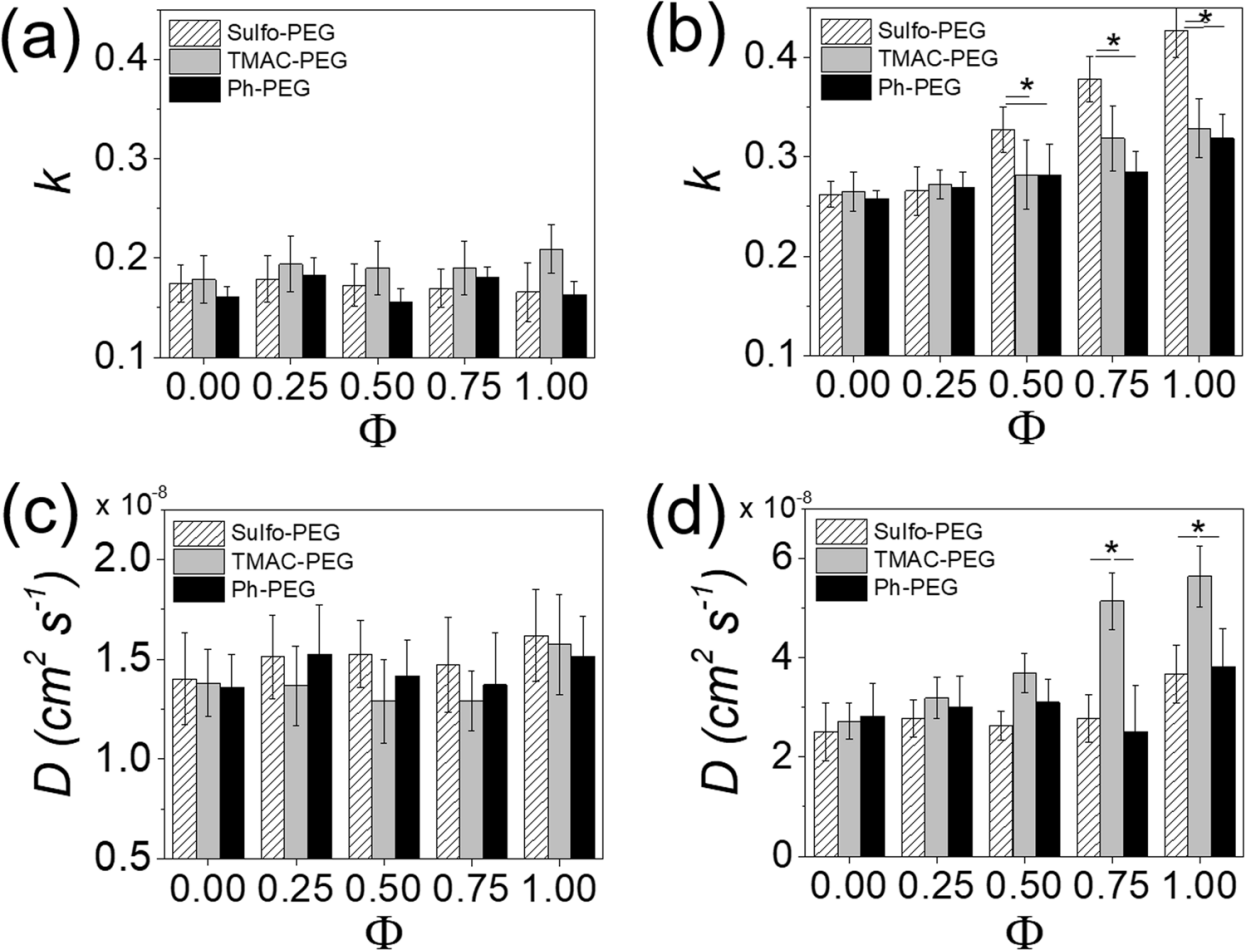
(**a**,**b**) Kinetic rate constants (*k*) and (**c**,**d**) diffusion coefficients (*D*) of albumin release from Sulfo-PEG, TMAC-PEG and Ph-PEG hydrogels, obtained by fitting the release profiles with Eq. (1) and Eq. (2), respectively. (*p < 0.05) (**a**,**c**) 10% PEGDA and 2% PEGMA, (**b**,**d**) 6% PEGDA and 6% PEGMA.

The albumin release from PEG hydrogels at different PEGMA concentrations were further evaluated to assess the effect of crosslinking density on the protein release (Fig. 6, see Supplementary Figs S7 and S8). When the PEGMA concentration was lowered to 2% (w/v) (PEGDA concentration increased to 10% (w/v)), the *k* and *D* values were generally low for all hydrogel types (Fig. 6a and c). In addition, there was also negligible effect of Φ on the *k* values. This result suggests that albumin release was significantly hindered by the diminished porosity of the hydrogels at higher crosslinking density, and as a result the effect of charged groups on albumin release became minimal. When the PEGMA concentration was increased to 6% (w/v) (PEGDA concentration decreased to 6% (w/v)), there was a substantial increase in *k* and *D* values with Φ for all types of hydrogels (Fig. 6b and d). The albumin release from Sulfo-PEG hydrogels was larger than other hydrogels, as expected. But there were smaller increases in albumin release from TMAC-PEG hydrogels and Ph-PEG hydrogels with Φ, despite the increasing positive charge and hydrophobicity. This is due to the increased porosity of the hydrogels, as evidenced by the swelling ratio shown in Fig. 4, facilitating the protein release from the hydrogels and thereby diminishing the effect of functional pendant groups.

#### Insulin

Insulin, a hormone produced by beta cells in pancreatic islets, regulates the glucose level in our body. It is the de facto glucose-lowering drug for diabetes, and biomaterial-based delivery systems have been widely investigated for its controlled release^34^. It has a pI value of 5.3, thus possessing negative charge at physiological pH, much like albumin. But since insulin (5.8 kDa) is much smaller than albumin (66 kDa), it was hypothesized that the level of control of insulin release from the charged PEG hydrogels would be different from that of albumin.

**Figure 7:**
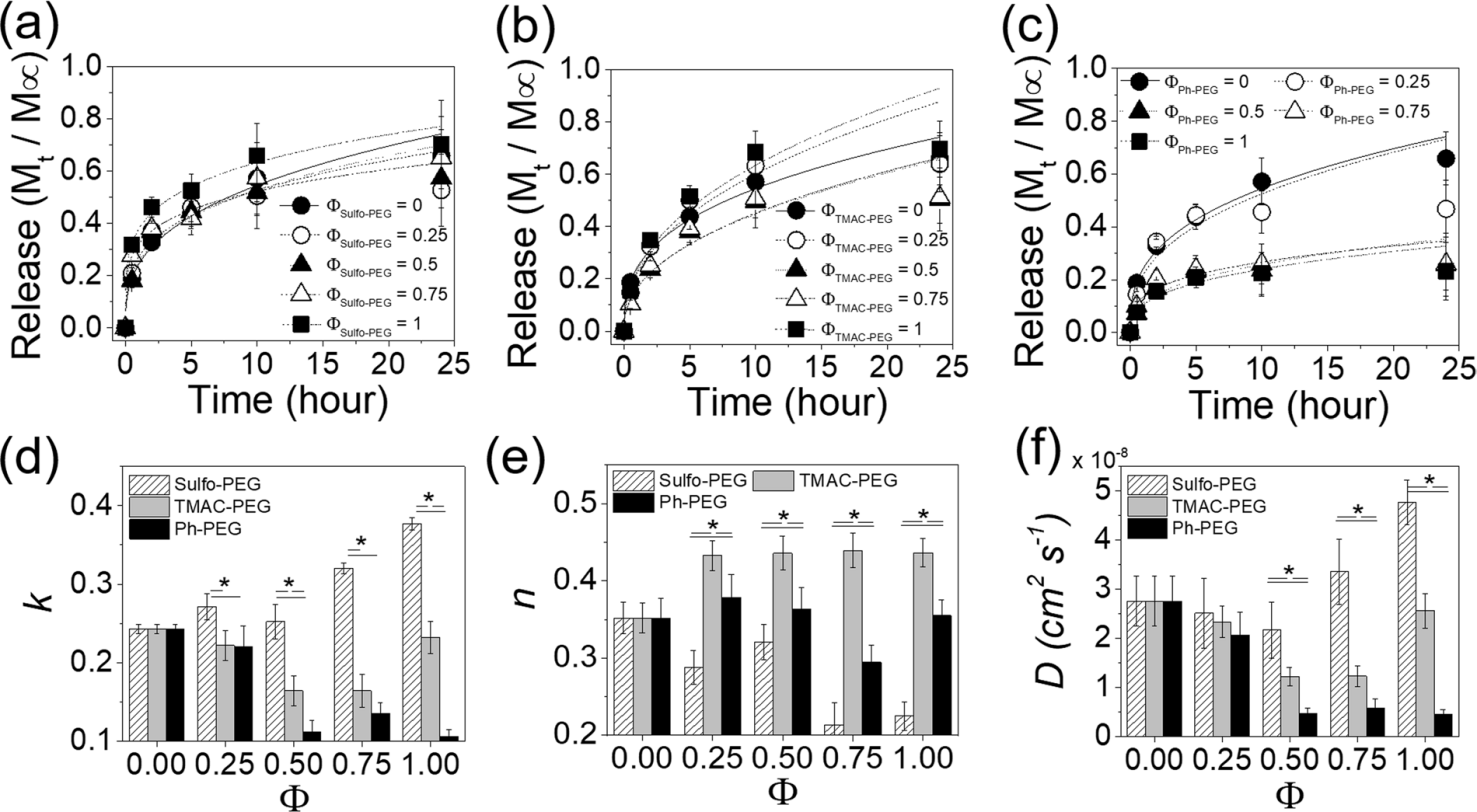
The release profiles of insulin from (**a**) Sulfo-PEG hydrogels, (**b**) TMAC-PEG hydrogels and (**c**) Ph-PEG hydrogels. (**d**) The kinetic rate constants (*k*) and (**e**) exponents (*n*) obtained by fitting the curves with Eq. (1). (**f**) Diffusion coefficients (*D*) were obtained by fitting the curves with Eq. (2). (*p < 0.05) The PEGDA and PEGMA concentrations of the hydrogels were 8% and 4%, respectively.

The concentration of PEGMA was first kept at 4% (w/v) (PEGDA concentration of 8% (w/v)). The *k* values of insulin in Sulfo-PEG hydrogels began to increase significantly when Φ was increased above 0.5, unlike the albumin whose release was promoted below 0.5 (Fig. 7a and d). Since there is less positive charge on insulin than albumin due to higher pI, more negative charges may have been needed to facilitate the release. It is also possible that the smaller size of insulin likely conferred more interaction with the polymeric network, requiring more negative charges to facilitate the release.

More significantly, the *k* values of insulin in TMAC-PEG hydrogels decreased above Φ = 0.5, demonstrating the increased positive charge helped delay the release of insulin, more so than albumin despite less negative charge (Fig. 7b and d). The smaller insulin likely was better able to interact with TMAC groups than bulkier albumin. The release from hydrophobic Ph-PEG hydrogels also showed significant decrease with Φ, demonstrating the increased number of phenyl groups was able to undergo extensive hydrophobic interaction with insulin, thereby delaying the release. Compared to albumin, less negative charge and smaller size of insulin likely contributed to this phenomenon.

Like albumin, the exponents (*n*) of insulin release from the hydrogels were all below 0.5, indicating the quasi-Fickian release with greater initial release (Fig. 7e). However, the *n* values from Sulfo-PEG hydrogels were significantly lower than those of TMAC-PEG and Ph-PEG hydrogels, which further highlight the much accelerated release of insulin from the Sulfo-PEG hydrogels as compare with other hydrogels, likely attributed to the smaller size compared to albumin. In addition, similar to the albumin release, the changes in diffusion coefficients (*D*) calculated from fitting with Eq. (2) with Φ and type of pendant chains were generally in line with the *k* values shown in Fig. 7d obtained from Eq.(1) (Fig. 7f, see Supplementary Fig. S9).

**Figure 8:**
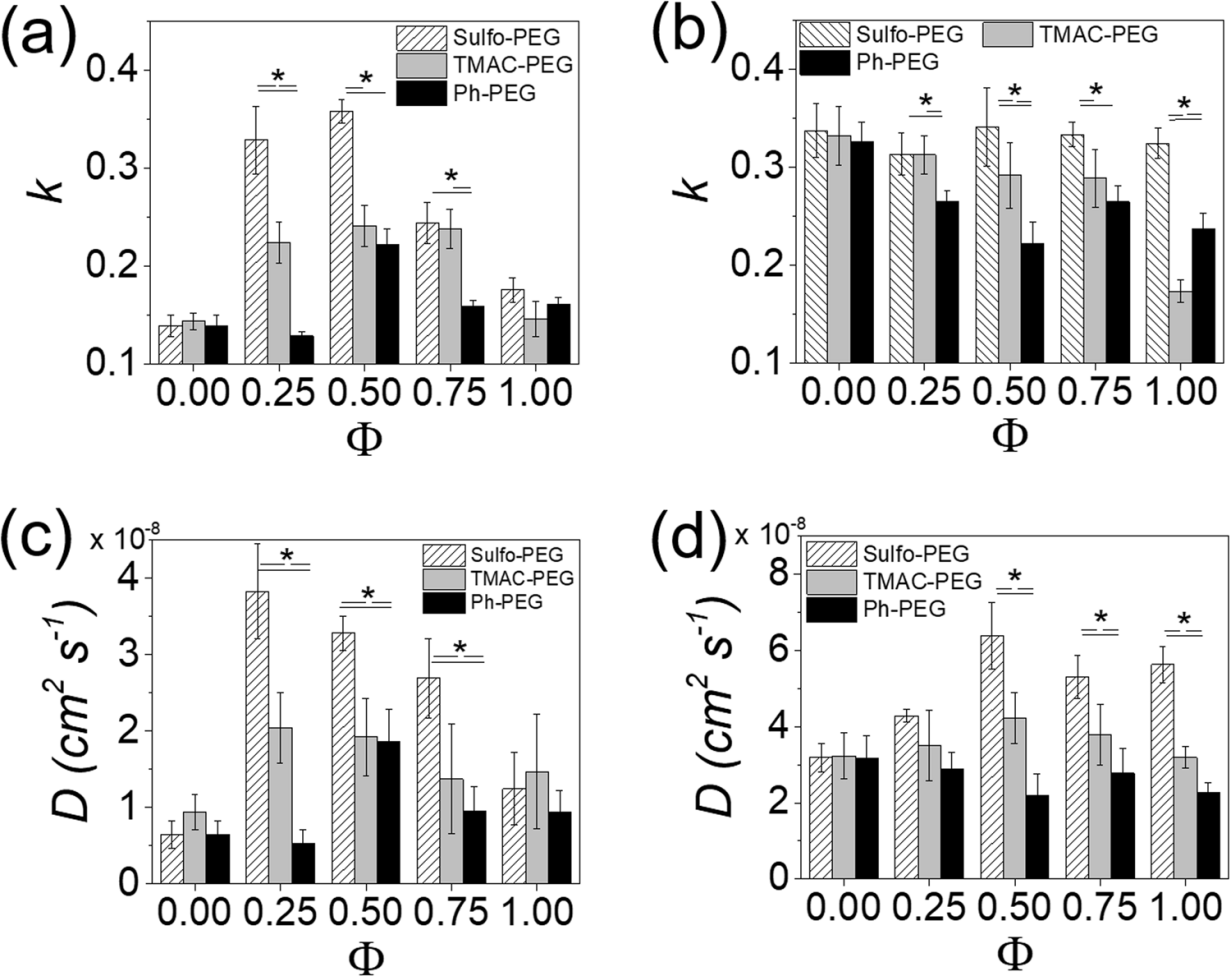
(**a**,**b**) Kinetic rate constants (*k*) and (**c**,**d**) diffusion coefficients (*D*) of insulin release from Sulfo-PEG, TMAC-PEG and Ph-PEG hydrogels, obtained by fitting the release profiles with Eq. (1) and Eq. (2), respectively. (*p < 0.05) (**a**,**c**) 10% PEGDA and 2% PEGMA, (**b**,**d**) 6% PEGDA and 6% PEGMA.

As similarly done with albumin, the insulin release from hydrogels were evaluated at different PEGMA concentrations to assess the effect of crosslinking density (Fig. 8, see Supplementary Figs S9 and S10). Interestingly, when the PEGMA concentration was reduced to 2% (w/v) (increasing PEGDA concentration to 10% (w/v)), the *k* and *D* values of insulin from Sulfo-PEG hydrogels showed significant increase with Φ up to 0.5, but decreased with Φ afterwards (Fig. 8a and c). Similarly, to lesser extent, the release profiles of TMAC-PEG hydrogels and Ph-PEG hydrogels followed the similar patterns. This biphasic behavior was not shown for albumin at the same PEGMA concentration in Fig. 6a, in which the overall release was diminished, and suggested that there is an optimal level of diffusion of smaller protein in a more crosslinked network. The initial increase in *k* values with Φ up to 0.5 could possibly be a result of insulin, which is much smaller than albumin, being able to diffuse from the hydrogels even through diminished porosity at higher crosslinking density via pressure-driven flow enhancement in confined spaces^35,36^. However, further increase in Φ likely reduced the flow enhancement, as the increased functional groups facilitated the increased porosity via the polymeric chain relaxation, eliminating the pressure gradient within the hydrogel. This interesting aspect of protein release in confined polymeric networks warrants further investigation in future studies.

For the hydrogels with higher PEGMA concentration of 6% (w/v) (the PEGDA concentration of 6% (w/v)), the overall *k* and *D* values were much larger than those at lower PEGMA concentrations, as expected, due to lower crosslinking density (Fig. 8b and d). The *k* values of Sulfo-PEG hydrogels remained high at all Φ, suggesting the increased negative charge density did not contribute to the release rates as they were already high. On the other hand, the *k* values of TMAC-PEG hydrogels decreased with Φ, demonstrating the enhanced electrostatic interaction with insulin by the increased positive charge density helped delay the release. The increased hydrophobic interaction by increasing Φ of Ph-PEG hydrogels also resulted in decreasing *k* values. Interestingly, the increase in *D* for Sulfo-PEG hydrogels with Φ was larger, and the difference between Sulfo-PEG, TMAC-PEG, and Ph-PEG at a given Φ was also much larger than *k* values shown in Fig. 8b (Fig. 8d). This was due to the overestimation by the Fickian diffusion model, which was accentuated by the larger initial drug release for hydrogels with lower crosslinking density. Regardless, the *D* values further demonstrated the greater release rates from Sulfo-PEG hydrogels. Taken together, the insulin release was more heavily influenced by the changes in charge density of PEG hydrogels with functional pendant chains, which suggested that insulin was better able to interact with the functional groups owing to the smaller size, as compared to albumin.

#### Trypsin

**Figure 9:**
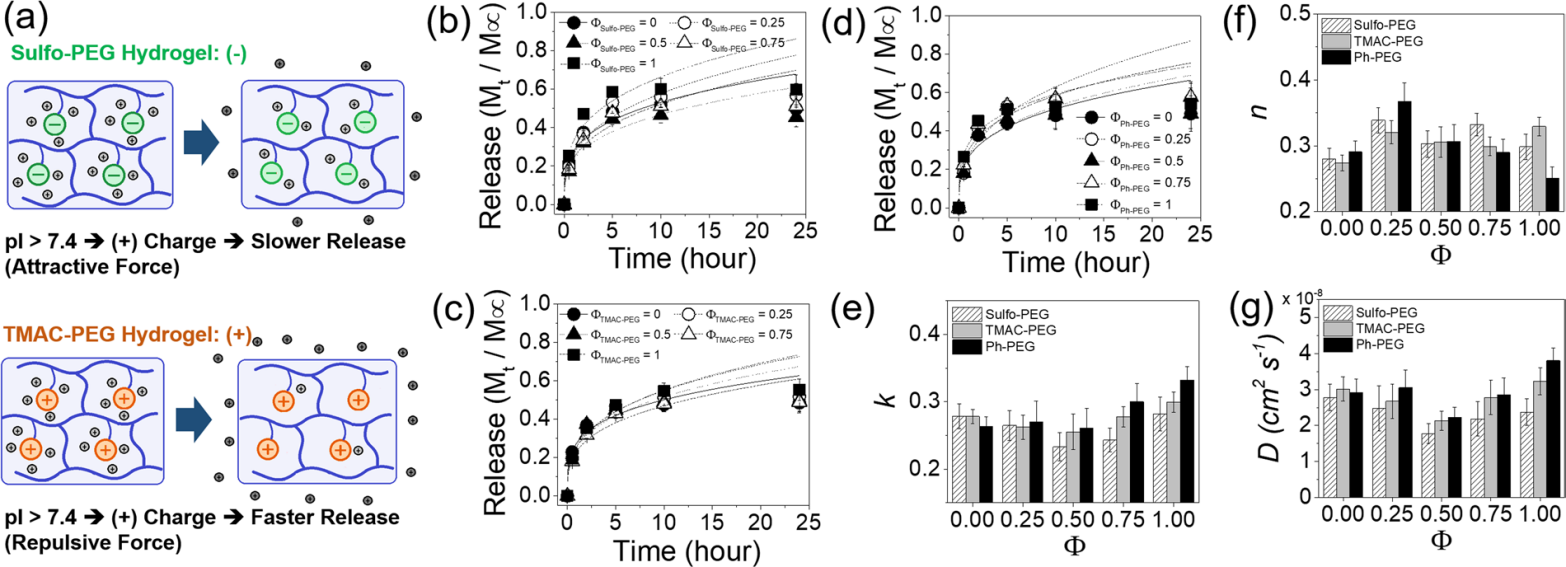
(**a**) Protein with pI higher than 7.4 becomes positively charged, and thus expected to have a greater release rate in TMAC-PEG hydrogels via repulsion than Sulfo-PEG hydrogels. The release profiles of trypsin from (**b**) Sulfo-PEG hydrogels, (**c**) TMAC-PEG hydrogels and (**d**) Ph-PEG hydrogels. (**e**) The kinetic rate constants (*k*) and (**f**) exponents (*n*) were values obtained by fitting the curves with Eq. (1). (**g**) Diffusion coefficient (*D*) were obtained by fitting the curves with Eq. (2). The PEGDA and PEGMA concentrations of the hydrogels were 8% and 4%, respectively.

Trypsin is a well-known serine protease responsible for protein digestion in many mammalian species, and also widely used for breaking cell aggregates and detaching cells from culture substrates in routine biological experiments. Unlike albumin and insulin, trypsin has the pI value of 10.5, thus possessing positive charge at physiological pH. Therefore, trypsin encapsulated in Sulfo-PEG hydrogels was expected to show lower release rates than TMAC-PEG hydrogels (Fig. 9a).

The concentration of PEGMA was first kept at 4% (w/v) (PEGDA concentration of 8% (w/v)). The *k* and *D* values indicate that the trypsin release from Sulfo-PEG hydrogels was significantly reduced at all Φ, compared to albumin and insulin, demonstrating the increased negative charge density of Sulfo-PEG hydrogels was able to decrease the release rate of positively-charged trypsin (Fig. 9b,e and f, see Supplementary Fig. 11). However, the release rates of trypsin from TMAC-PEG hydrogels was not significantly enhanced by the increased positive charge via electrostatic repulsion as predicted (Fig. 9c and e). This result suggests the positive charge density conferred by TMAC groups was not strong enough to exert enough repulsive force to facilitate the release of trypsin, which was in part evidenced by the lower absolute zeta potential value of TMAC (0.11 mV) compared to Sulfo group (0.21 mV). The exponents, like albumin, were not significantly affected by the type of pendant chains in the hydrogels (Fig. 9f).

**Figure 10:**
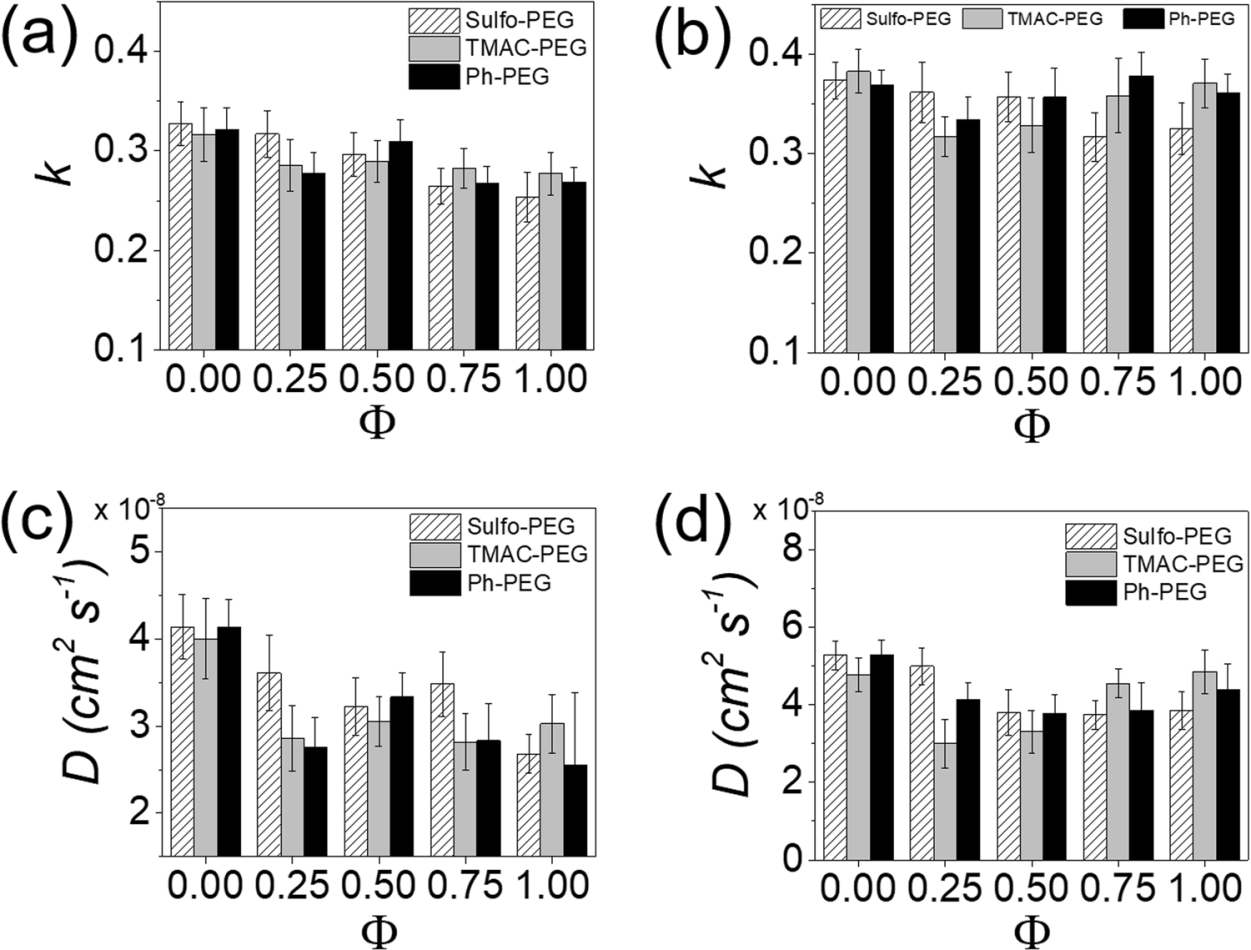
(**a**,**b**) Kinetic rate constants (*k*) and (**c**,**d**) diffusion coefficients (*D*) of trypsin release from Sulfo-PEG, TMAC-PEG and Ph-PEG hydrogels, obtained by fitting the release profiles with Eq. (1) and Eq. (2), respectively. (**a**) 10% PEGDA and 2% PEGMA, (**b**) 6% PEGDA and 6% PEGMA.

Similarly, for the hydrogels at other PEGMA concentrations, the *k* and *D* values of trypsin in Sulfo-PEG hydrogels were significantly reduced as compared to albumin and insulin, whereas those in TMAC-PEG hydrogels did not significantly change with Φ (Fig. 10, see Supplementary Figs S11 and S12). The *k* values in Ph-PEG hydrogels, regardless of the crosslinking density of the hydrogels, were also not substantially affected by Φ. It is possible that the range of physical properties tuned by the TMAC-PEG hydrogels and Ph-PEG hydrogels used in this study were not enough to significantly influence the release rate of trypsin.

## Methods

### Synthesis of poly(ethylene glycol) dimethacrylate (PEGDA)

PEGDA was synthesized following a previously published method^30^. Briefly, poly(ethylene glycol) (PEG, average M_W_ of 2,000 g mol^−1^, Sigma Aldrich) and triethylamine (Sigma Aldrich) were first dissolved in dichloromethane. Methacryloyl chloride (Sigma Aldrich) was added dropwise to the mixture, and continuously stirred for 24 hours under dry N_2_ gas. The molar ratio of PEG, methacryloyl chloride and triethylamine was 1:4:5. The insoluble salt formed during the reaction was filtered out, and excess diethyl ether was added to the mixture to precipitate the product. The product was obtained by filtration, washed twice with diethyl ether, and dried under vacuum.

### Synthesis of heterobifunctional poly(ethylene glycol) monomethacrylate (PEGMA)

Heterobifunctional PEGMA having a functional end group was developed by two-step synthesis: (1) the ring-opening anionic polymerization of ethylene oxide to synthesize heterobifunctional PEG, followed by (2) the conjugation of methacrylate onto the hydroxyl group on the PEG (Fig. 1b)^30,37,38^.

Potassium naphthalenide, a strong base that activates the hydroxyl-based initiator to alkoxide, was prepared by dissolving molar equivalents of potassium and naphthalene (9 mmol each) in 20 mL of dry tetrahydrofuran (THF, Samchun Chemical, Korea) for 5 hours. The mixture became dark green with the formation of potassium naphthalenide. Hydroxyl-based initiator (11 mmol) was dissolved separately in 30 mL of dry THF, and the potassium naphthalenide mixture was slowly added and stirred for one hour, resulting in the alkoxide formation. 30 mL of ethylene oxide (EO, Asia Chemical, Korea) was then added to the mixture and continuously stirred for 48 hours at room temperature (note: the mixture and EO was chilled on ice to prevent evaporation during the mixing process). The entire process was done under dry N_2_ gas.

HCl (10 mmol) was added to stop and neutralize the reaction, from which the insoluble salt (KCl) being formed was filtered out. After condensing the mixture via rotary evaporation, the crude product was obtained by precipitation in excess diethyl ether. After washing with diethyl ether twice, the final product was obtained by filtration and drying under vacuum. The initiators used for synthesizing sodium sulfoethyl, trimethylammonium chloride, and phenyl-capped PEG, namely Sulfo-PEG, TMAC-PEG, and Ph-PEG, were sodium isethionate, choline chloride, and 2-phenylethanol (all purchased from Sigma Aldrich), respectively. The molecular weights (M_W_) of Sulfo-PEG, TMAC-PEG, and Ph-PEG, as determined from MALDI-TOF mass spectrometry (Ultraflex III, Bruker), were 2341, 2200, and 1770 g mol^−1^, respectively (see Supplementary Fig. S13). For Sulfo-PEG and TMAC-PEG, tetrahydrofuran and 2,5-dihydroxybenzoid acid (Sigma Aldrich) were used as solvent and matrix, respectively, while chloroform and dithranol (Sigma Aldrich) were used for Ph-PEG.

The conjugation of methacrylate was done using the same procedure for the synthesis of PEGDA. The molar ratio of heterobifunctional PEG, methacryloyl chloride and TEA here was 1:2:3, as there is only one hydroxyl group in a polymer chain.

The chemical structures of the heterobifunctional PEGMAs were analyzed with ^1^H-NMR and ^13^C-NMR spectra (400-MR DD2, Agilent) (see Supplementary Figs S1 and S2). The M_W_ of Sulfo-PEGMA, TMAC-PEGMA and Ph-PEGMA, determined by the ratio of integration peaks corresponding to methacrylate and ethylene oxide unit, were 3370, 3211, and 4120 g mol^−1^, respectively. The zeta potentials of sulfonate and trimethylammonium chloride linked PEG were determined at 0.1 wt% in 10 mM NaCl, pH 7 (Nano ZS, Malvern) (see Supplementary Fig. S3).

### Fabrication of hydrogels

A precursor solution was prepared by mixing PEGDA and PEGMA in various ratios, while keeping the overall concentration at 12% (w/v). The total PEGMA content consisted of ‘non-functional’ methoxy PEGMA (M_n_ 2000 g mol^−1^, Sigma Aldrich) and the heterobifunctional PEGMA. At a given PEGMA concentration, the fraction of heterobifunctional PEGMA was varied to control the physical properties of the pendant PEG chains. 10 μL of ammonium persulfate (1 M) and 1 μL of tetramethylethylenediamine as co-initiators were added to 1 mL of a precursor solution, and placed in a custom-made mold with 1 mm spacer to prepare disk-shaped hydrogels with a diameter of 8 mm. The hydrogel disks were incubated in phosphate buffered saline (PBS) at room temperature for 24 hours before mechanical characterization.

### Evaluation of mechanical properties of hydrogels

The mechanical properties of hydrogels were evaluated by calculating elastic moduli from stress-strain curves obtained from uniaxial compression experiments (Model 3343, Instron)^24,39,40^. Each hydrogel disk was compressed at a rate of 1 mm min^−1^. The elastic modulus was calculated from the slope of a stress-strain curve at the initial 10% strain region where the curve was linear.

### Evaluation of protein release from hydrogels

The effect of physical properties of hydrogels on drug release kinetics was evaluated by measuring time-dependent release profiles of proteins with varying pI values. Albumin (from human serum, Sigma Aldrich), trypsin (from bovine pancreas, Sigma Aldrich) and insulin (human recombinant, Sigma Aldrich) having pI values of 4.7, 5.3, and 10.5, respectively, were used as model proteins. Their zeta potentials were measured at −14.2 mV, −5.7 mV, and 22.1 mV, respectively (0.1 wt% in 10 mM NaCl, pH 7) (see Supplementary Fig. S1). The protein was first encapsulated into the hydrogel by dissolving the protein (2 mg mL^−1^) in a precursor solution prior to fabrication. The hydrogel disks were prepared as mentioned above. The hydrogel disks were incubated in PBS at 37 °C, and at each designated time point, the incubating media were collected and the protein concentrations were obtained using BCA^TM^ Protein Assay kit (Thermo Fisher), following the manufacturer’s instructions. The cumulative protein release profiles were fitted to the Ritger-Peppas equation,

1 $$\frac{{M}_{t}}{{M}_{\infty }}\,=\,k\,\cdot \,{t}^{n}$$

where *M*_*t*_ was the cumulative amount of protein released at a time, *t*, *M*_*∞*_ was the total amount of protein in the hydrogel, *k* was the kinetic rate constant, and *n* is the exponent related to the release mechanism^30,39,41^.

Alternatively, the cumulative protein release profiles were fitted to the following power-law equation following Fickian diffusion mechanism,

2 $$\frac{{M}_{t}}{{M}_{\infty }}\,=\,4{(\frac{Dt}{\pi {h}^{2}})}^{1/2}$$

where *D* was the diffusion coefficient and *h* was the thickness of the hydrogel disk samples^32,33,42^. This relationship generally fits well for the first 60% of the release due to the fixed time dependency (*t*^1/2^).

## Conclusion

In this study, various physical properties (i.e. charge density and hydrophobicity) of PEG hydrogels were controlled independent of the crosslinking density by presenting pendant PEG chains with characteristic end functional groups in order to control the release of proteins having different isoelectric points (pI). The PEG hydrogels were fabricated by crosslinking PEGDA with PEGMA which becomes pendant PEG chains. While keeping the PEGDA and PEGMA concentrations, the ratio of non-functional PEGMA and heterobifunctional PEGMA was varied to control the physical properties of the hydrogels without changing the crosslinking density nor the number of total pendant chains. Therefore, only the change in physical properties were expected to significantly influence the changes in protein release rates. Here, the heterobifunctional PEGMA with sulfonate (‘Sulfo-PEG’), trimethylammonium chloride (‘TMAC-PEG’), and phenyl group (‘Ph-PEG’) were synthesized to confer negative charge, positive charge, and hydrophobicity, respectively. The releases of albumin and insulin, whose pI values are lower than physiological pH, negatively charged, are more facilitated in Sulfo-PEG hydrogels due to electrostatic repulsion as compared to TMAC-PEG hydrogels capable of providing attractive force. In addition, the effect of charge type and density was more noticeable for insulin, likely due to its smaller size than albumin allowing more interactions with the functional groups. On the other hand, the release of trypsin, having positive charge due to higher pI value than physiological pH, was significantly diminished in Sulfo-PEG hydrogels via repulsive force. These results demonstrate that the PEG hydrogels with pendant functional groups could be successfully utilized as vehicles for refined control of protein release for biomedical applications.

### Data availability

All data generated or analysed during this study are included in this published article (and its Supplementary Information files).

## Electronic supplementary material

Supplementary Information

## References

[1] Vermonden T, Censi R, Hennink WE (2012). Hydrogels for protein delivery. Chem. Rev..

[2] Wang C, Varshney RR, Wang D-A (2010). Therapeutic cell delivery and fate control in hydrogels and hydrogel hybrids. Adv. Drug Deliv. Rev..

[3] Drury JL, Mooney DJ (2003). Hydrogels for tissue engineering: scaffold design variables and applications. Biomaterials.

[4] Schmidt JJ, Rowley J, Kong HJ (2008). Hydrogels used for cell-based drug delivery. J. Biomed. Mater. Res. A.

[5] Ruoslahti E (1996). RGD and other recognition sequences for integrins. Annu. Rev. Cell Dev. Biol..

[6] Zhu J (2010). Bioactive modification of poly(ethylene glycol) hydrogels for tissue engineering. Biomaterials.

[7] Lin C-C, Anseth KS (2009). PEG Hydrogels for the Controlled Release of Biomolecules in Regenerative Medicine. Pharma. Res..

[8] Weber LM, Lopez CG, Anseth KS (2009). Effects of PEG hydrogel crosslinking density on protein diffusion and encapsulated islet survival and function. J. Biomed. Mater. Res. A.

[9] Hoare TR, Kohane DS (2008). Hydrogels in drug delivery: progress and challenges. Polymer.

[10] Anseth KS, Bowman CN, Brannon-Peppas L (1996). Mechanical properties of hydrogels and their experimental determination. Biomaterials.

[11] Cha C, Kim SY, Cao L, Kong H (2010). Decoupled control of stiffness and permeability with a cell-encapsulating poly(ethylene glycol) dimethacrylate hydrogel. Biomaterials.

[12] Cruise GM, Scharp DS, Hubbell JA (1998). Characterization of permeability and network structure of interfacially photopolymerized poly(ethylene glycol) diacrylate hydrogels. Biomaterials.

[13] Fefelova NA, Nurkeeva ZS, Mun GA, Khutoryanskiy VV (2007). Mucoadhesive interactions of amphiphilic cationic copolymers based on [2-(methacryloyloxy)ethyl]trimethylammonium chloride. Int. J. Pharm..

[14] Huynh DP, Im GJ, Chae SY, Lee KC, Lee DS (2009). Controlled release of insulin from pH/temperature-sensitive injectable pentablock copolymer hydrogel. J. Control. Release.

[15] Lee W-F, Yeh P-L (1999). Thermoreversible hydrogels. VIII. Effect of a zwitterionic monomer on swelling behaviors of thermosensitive hydrogels copolymerized by N-isopropylacrylamide with N,N′-dimethyl (acrylamidopropyl) ammonium propane sulfonate. J. Appl. Polym. Sci..

[16] Matsusaki M, Akashi M (2005). Novel functional biodegradable polymer IV:  pH-sensitive controlled release of fibroblast growth factor-2 from a poly(γ-glutamic acid)-sulfonate matrix for tissue engineering. Biomacromolecules.

[17] Vinogradov SV, Bronich TK, Kabanov AV (2002). Nanosized cationic hydrogels for drug delivery: preparation, properties and interactions with cells. Adv. Drug Deliv. Rev..

[18] Yu L, Zhang Z, Ding J (2012). *In vitro* degradation and protein release of transparent and opaque physical hydrogels of block copolymers at body temperature. Macromol. Res..

[19] Bae MS (2016). Development of novel photopolymerizable hyaluronic acid/heparin-based hydrogel scaffolds with a controlled release of growth factors for enhanced bone regeneration. Macromol. Res..

[20] Zustiak SP, Leach JB (2010). Hydrolytically degradable poly(ethylene glycol) hydrogel scaffolds with tunable degradation and mechanical properties. Biomacromolecules.

[21] Peppas NA, Keys KB, Torres-Lugo M, Lowman AM (1999). Poly(ethylene glycol)-containing hydrogels in drug delivery. J. Control. Release.

[22] Alexander A, Ajazuddin, Khan J, Saraf S, Saraf S (2013). Poly(ethylene glycol)–poly(lactic-co-glycolic acid) based thermosensitive injectable hydrogels for biomedical applications. J. Control. Release.

[23] Lee W-F, Chen Y-C (2004). Effect of bentonite on the physical properties and drug-release behavior of poly(AA-co-PEGMEA)/bentonite nanocomposite hydrogels for mucoadhesive. J. Appl. Polym. Sci..

[24] Jang J, Hong J, Cha C (2017). Effects of precursor composition and mode of crosslinking on mechanical properties of graphene oxide reinforced composite hydrogels. J. Mech. Behav. Biomed. Mater..

[25] Sohn SS, Revuri V, Nurunnabi M, Kwak KS, Lee Y-k (2016). Biomimetic and photo crosslinked hyaluronic acid/pluronic F127 hydrogels with enhanced mechanical and elastic properties to be applied in tissue engineering. Macromol. Res..

[26] Lai F, Li H (2010). Modeling of effect of initial fixed charge density on smart hydrogel response to ionic strength of environmental solution. Soft Matter.

[27] Bueno VB, Bentini R, Catalani LH, Petri DFS (2013). Synthesis and swelling behavior of xanthan-based hydrogels. Carbohydr. Polym..

[28] Serra L, Doménech J, Peppas NA (2006). Drug transport mechanisms and release kinetics from molecularly designed poly(acrylic acid-g-ethylene glycol) hydrogels. Biomaterials.

[29] Kragh-Hansen U (1981). Molecular aspects of ligand binding to serum albumin. Pharmacol. Rev..

[30] Kim S, Lee K, Cha C (2016). Refined control of thermoresponsive swelling/deswelling and drug release properties of poly(N-isopropylacrylamide) hydrogels using hydrophilic polymer crosslinkers. J. Biomater. Sci. Polym. Ed..

[31] Cevik O, Gidon D, Kizilel S (2015). Visible-light-induced synthesis of pH-responsive composite hydrogels for controlled delivery of the anticonvulsant drug pregabalin. Acta Biomater..

[32] Ferrero C, Massuelle D, Doelker E (2010). Towards elucidation of the drug release mechanism from compressed hydrophilic matrices made of cellulose ethers. II. Evaluation of a possible swelling-controlled drug release mechanism using dimensionless analysis. J. Control. Release.

[33] Fu Y, Kao WJ (2010). Drug release kinetics and transport mechanisms of non-degradable and degradable polymeric delivery systems. Expert Opin. Drug Deliv..

[34] Chaturvedi K, Ganguly K, Nadagouda MN, Aminabhavi TM (2013). Polymeric hydrogels for oral insulin delivery. J. Control. Release.

[35] Holt JK (2006). Fast mass transport through sub-2-nanometer carbon nanotubes. Science.

[36] Thomas JA, McGaughey AJH (2008). Reassessing fast water transport through carbon nanotubes. Nano Lett..

[37] Akiyama Y, Nagasaki Y, Kataoka K (2004). Synthesis of heterotelechelic poly(ethylene glycol) derivatives having α-benzaldehyde and ω-pyridyl disulfide groups by ring opening polymerization of ethylene oxide using 4-(diethoxymethyl)benzyl alkoxide as a novel initiator. Bioconjugate Chem..

[38] Cammas S, Nagasaki Y, Kataoka K (1995). Heterobifunctional poly(ethylene oxide): Synthesis of a-methoxy-w-amino and a-hydroxy-w-amino PEOs with the same molecular weights. Bioconjugate Chem..

[39] Cha C, Kohman RE, Kong H (2009). Biodegradable polymer crosslinker: Independent control of stiffness, toughness, and hydrogel degradation rate. Adv. Funct. Mater..

[40] Kim S, Sim SB, Lee K, Cha C (2017). Comprehensive examination of mechanical and diffusional effects on cell behavior using a decoupled 3d hydrogel system. Macromol. Biosci..

[41] Cha C, Jeong JH, Kong H (2015). Poly(ethylene glycol)-poly(lactic-co-glycolic acid) core–shell microspheres with enhanced controllability of drug encapsulation and release rate. J. Biomater. Sci. Polym. Ed..

[42] Siepmann J, Peppas NA (2012). Modeling of drug release from delivery systems based on hydroxypropyl methylcellulose (HPMC). Adv. Drug Deliv. Rev..

