# Supplementary material for: Modulation of functional pendant chains within poly(ethylene glycol) hydrogels for refined control of protein release
Source: Sci Rep. 2018 Mar 12;8:4315. doi: 10.1038/s41598-018-22249-1 (PMC5847582; doi:10.1038/s41598-018-22249-1)
Supplement: Supplementary file 1 — Supplementary Information [file 41598_2018_22249_MOESM1_ESM.pdf]

Supplementary Information

Modulation of functional pendant chains within poly(ethylene glycol) hydrogels for refined control of protein release

Mirae Kim[1], Chaenyung Cha[1,*]

[1]School of Materials Science and Engineering, Ulsan National Institute of Science and Technology, Ulsan 44919, South Korea

[*]Corresponding Author
Email: ccha@unist.ac.kr, Tel: +82-52-217-5328, Fax: +82-52-217-2019

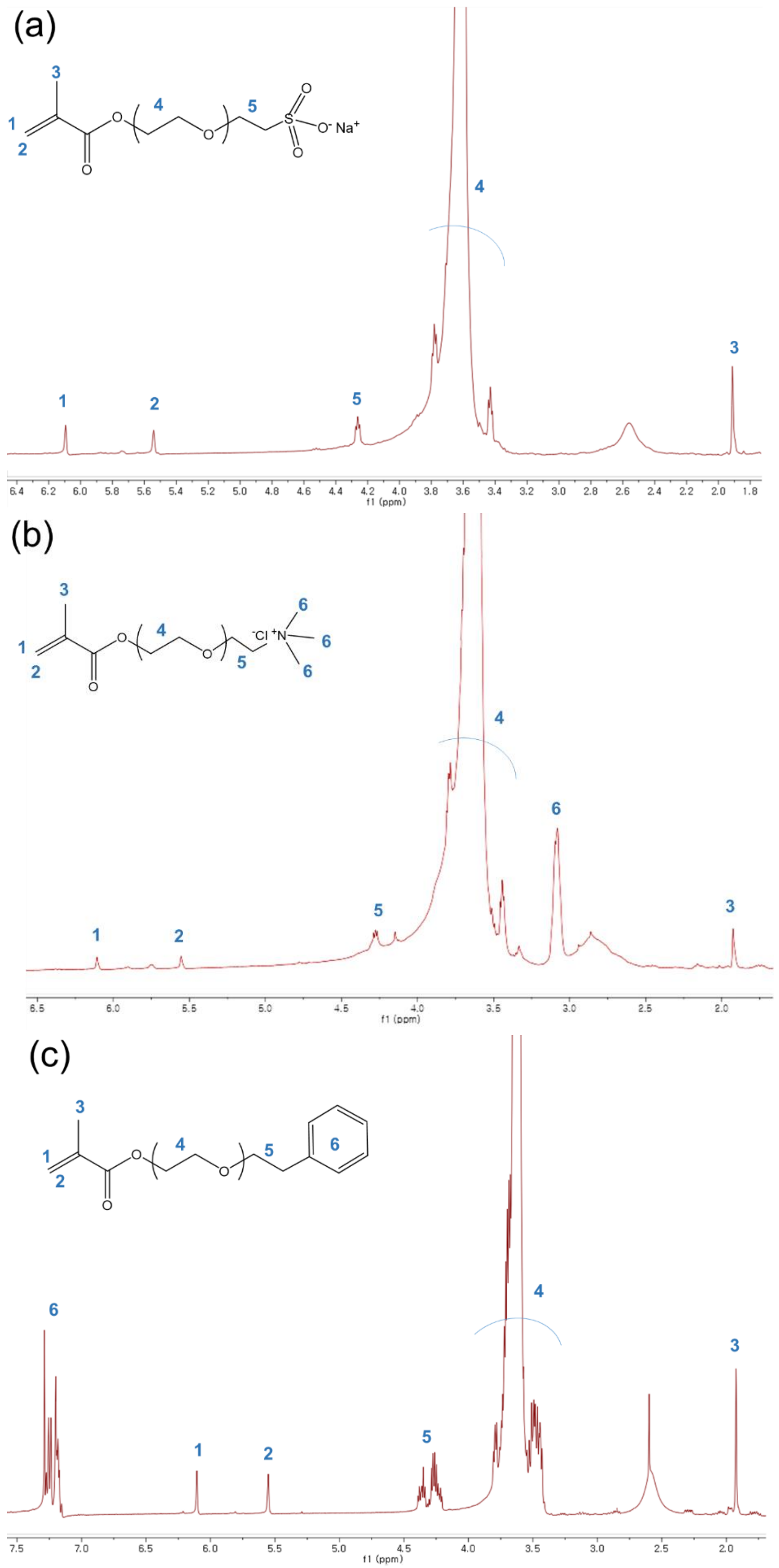

FIGURE S1. ^{1}H-NMR spectra of (a) Sulfo-PEG, (b) TMAC-PEG, and (c) Ph-PEG.

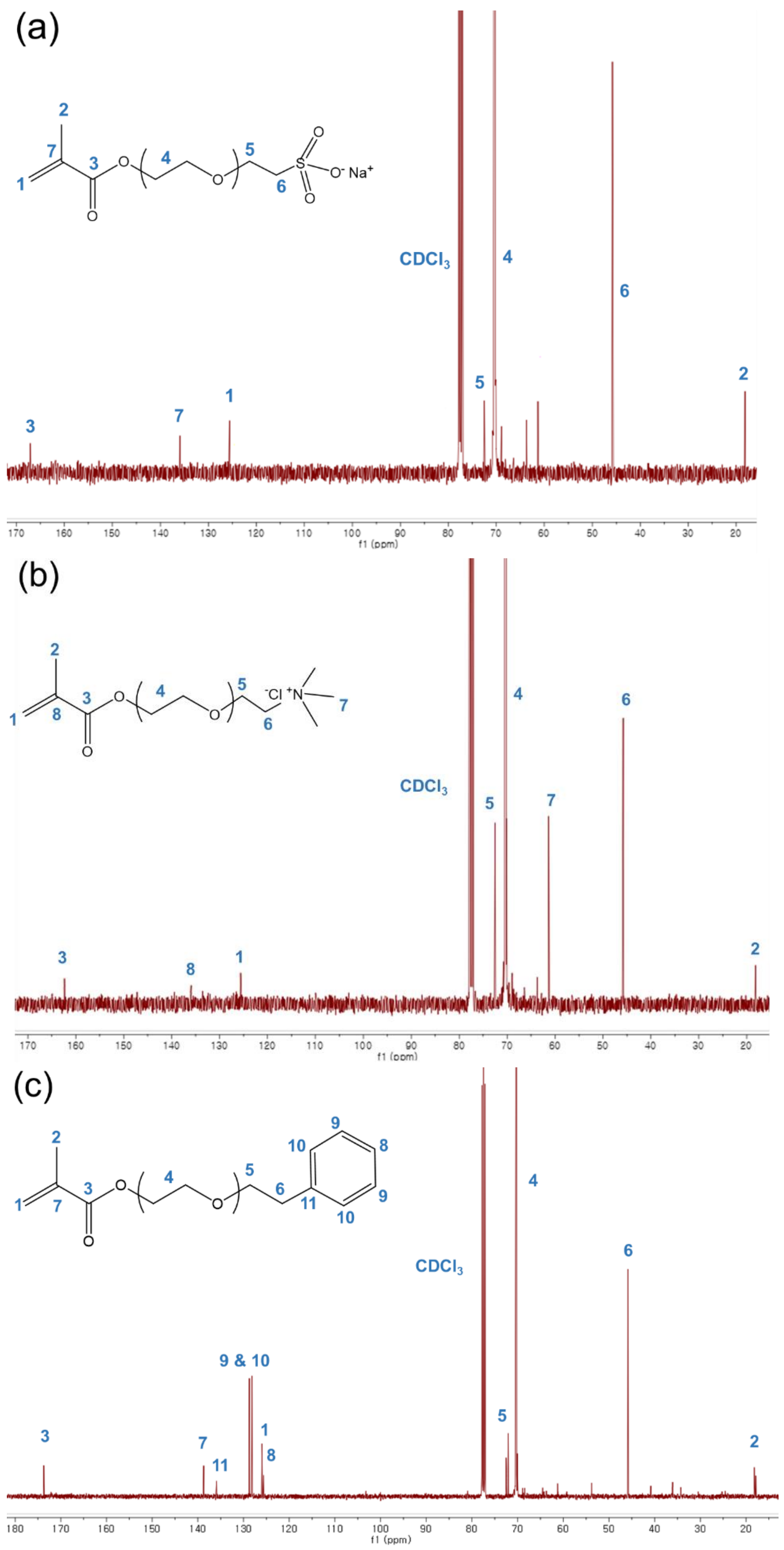

FIGURE S2. ^{13}C-NMR spectra of (a) Sulfo-PEG, (b) TMAC-PEG, and (c) Ph-PEG.

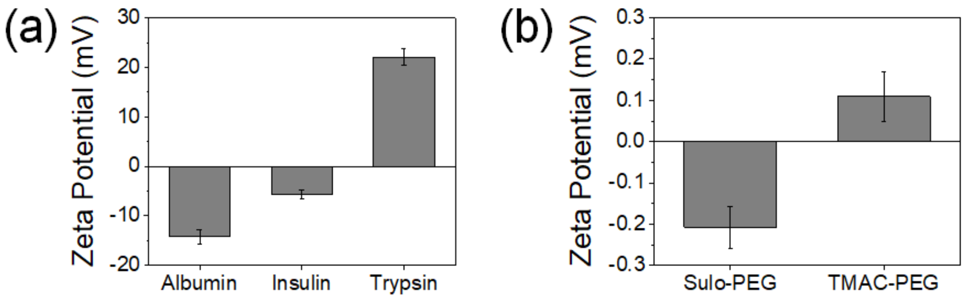

FIGURE S3. Zeta potential values of (a) proteins (albumin, insulin, and trypsin) and (b) Sulfo-PEGMA and TMAC-PEGMA, measured at 0.1 % (w/v) in 10 mM NaCl.

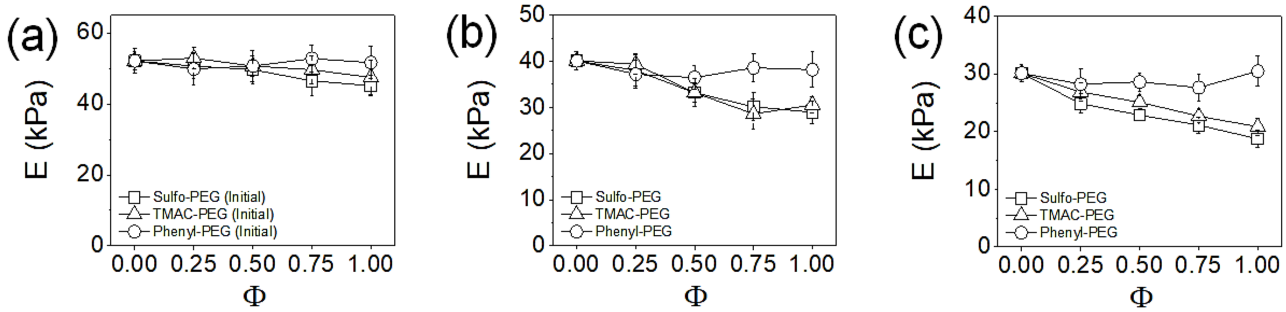

FIGURE S4. Elastic moduli (*E*) of Sulfo-PEG, TMAC-PEG, and Ph-PEG hydrogels measured right after fabtication ('initial moduli'). The PEGDA and PEGMA concentrations of the hydrogels were (a) 10 % and 2 %, (b) 8 % and 4 %, and (c) 6 % and 6 %.

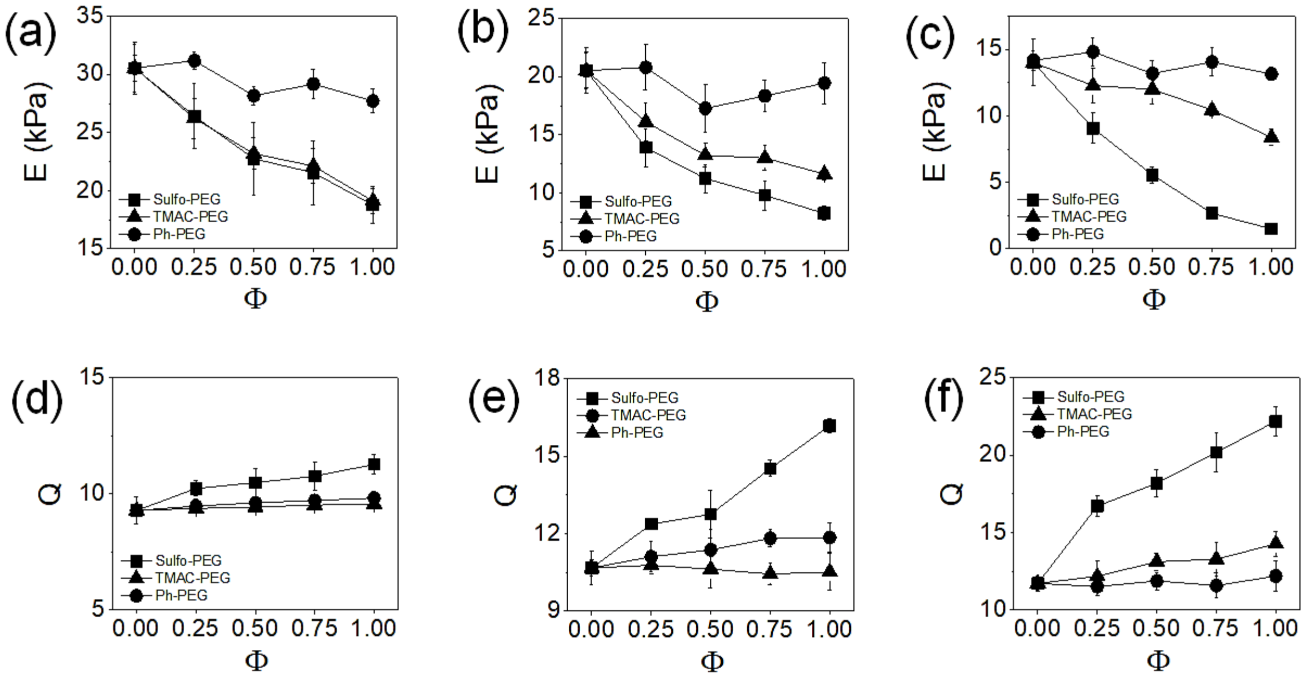

FIGURE S5. (a-c) Elastic moduli (*E*) and (d-e) swelling ratios (*Q*) of Sulfo-PEG, TMAC-PEG, and Ph-PEG hydrogels. The PEGDA and PEGMA concentrations of the hydrogels were (a,d) 10 % and 2 %, (b,e) 8 % and 4 %, and (c,f) 6 % and 6 %. *E* and *Q* were measured after 1 day of incubation in PBS.

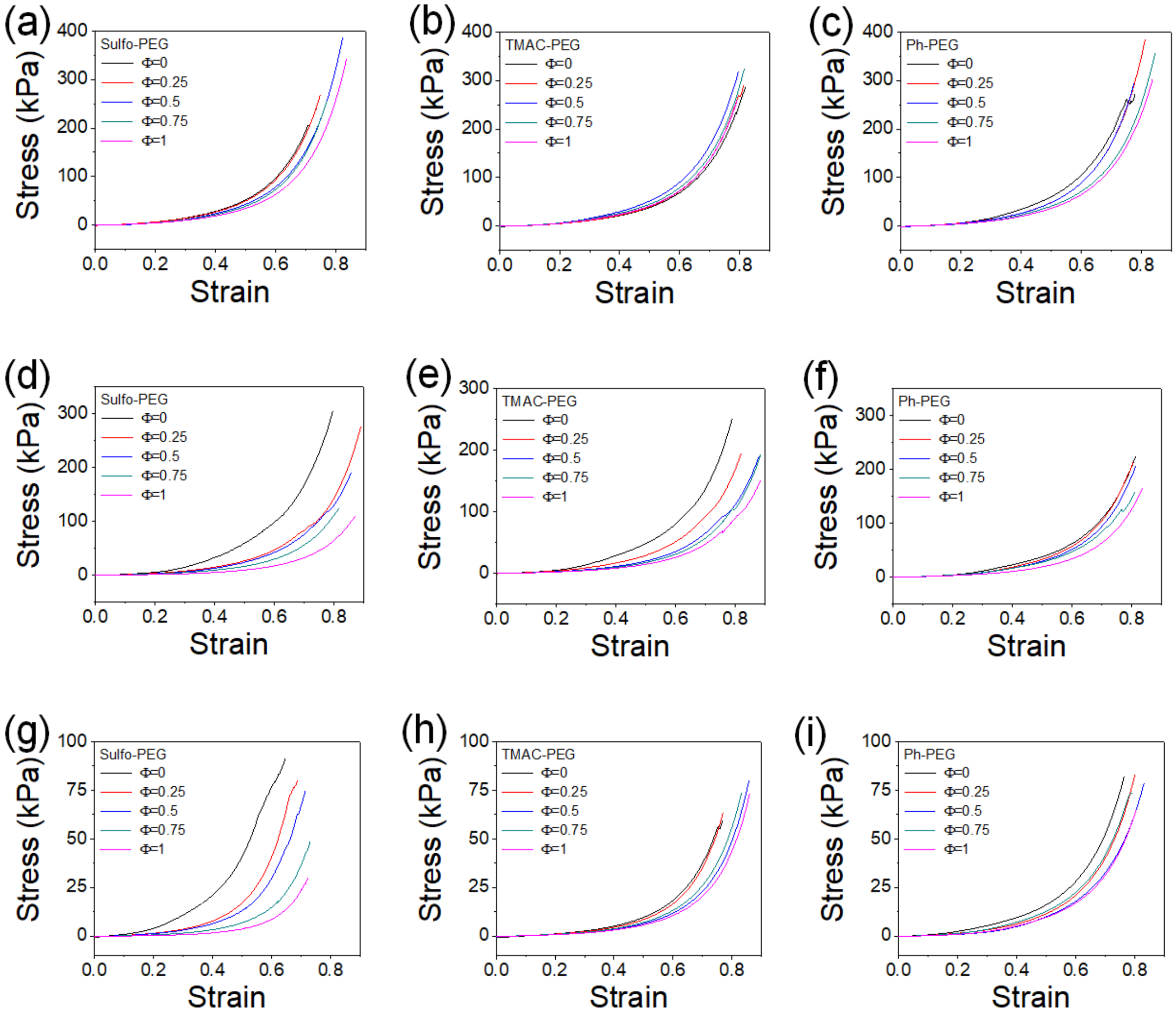

FIGURE S6. Strain-stress curves of (a, d, g) Sulfo-PEG, (b, e, h) TMAC-PEG, and (c, f, i) Ph-PEG hydrogels obtained by uniaxial compression. The PEGDA and PEGMA concentrations of the hydrogels were (a-c) 10 % and 2 %, (d-f) 8 % and 4 %, and (g-i) 6 % and 6 %.

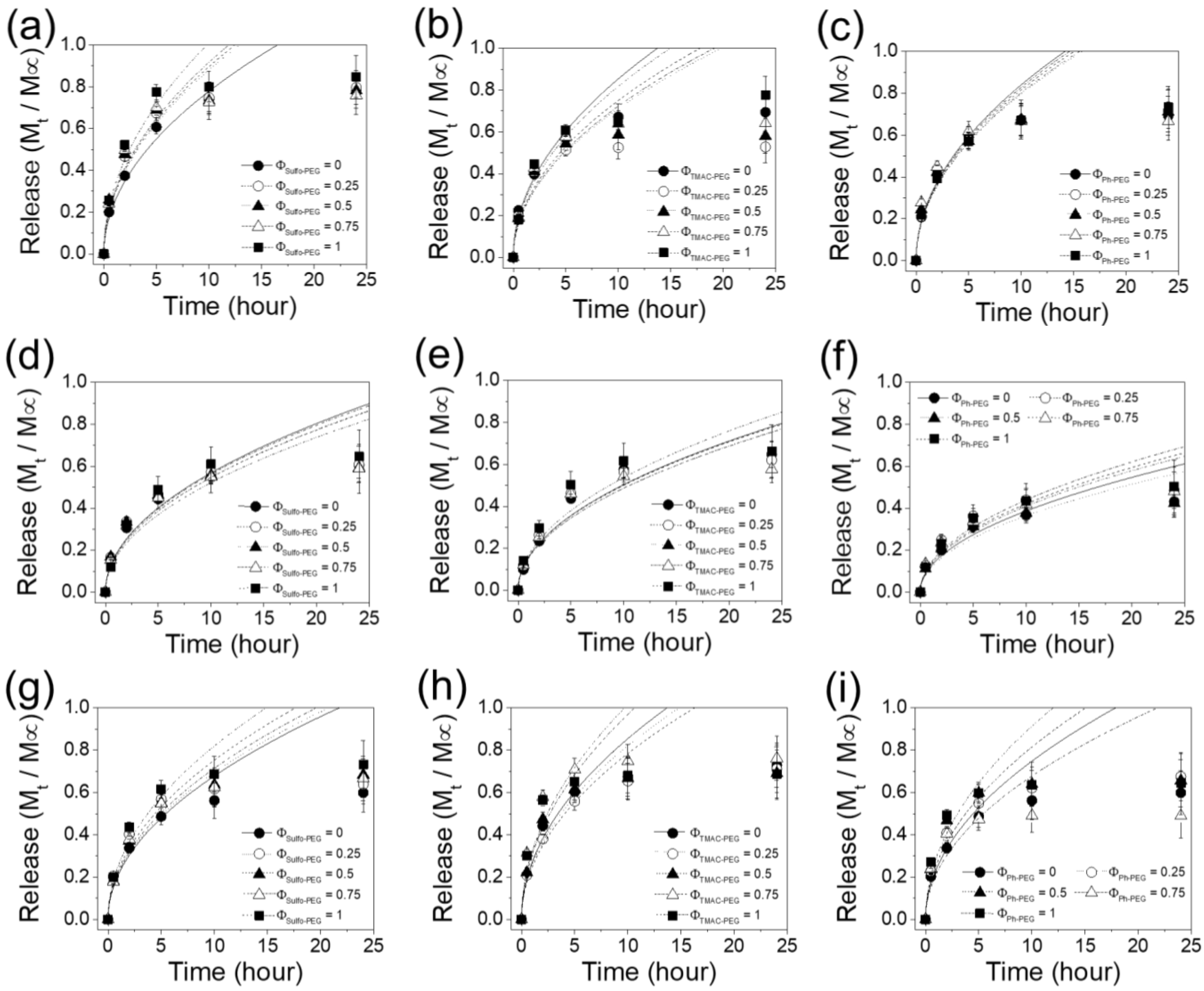

FIGURE S7. Release profiles of albumin from (a, d, g) Sulfo-PEG hydrogels, (b, e, h) TMAC-PEG hydrogels, and (c, f, i) Ph-PEG hydrogels. The profiles were fitted with Eq. (2). The PEGDA and PEGMA concentrations of the hydrogels were (a-c) 8 % and 4 %, (d-f) 10 % and 2 %, and (g-i) 6 % and 6 %.

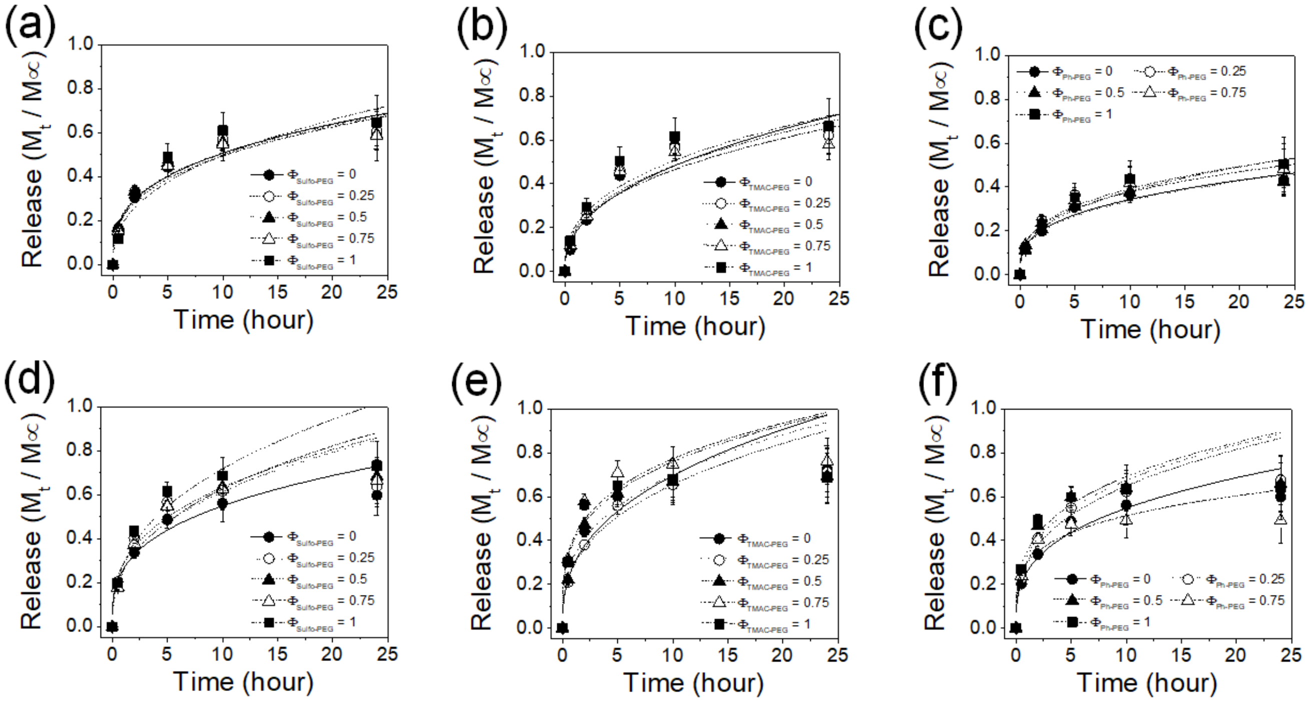

FIGURE S8. Release profiles of albumin from (a, d) Sulfo-PEG hydrogels, (b, e) TMAC-PEG hydrogels, and (c, f) Ph-PEG hydrogels. The profiles were fitted with Eq. (1). (a-c) 10% PEGDA and 2% PEGMA, (d-f) 6% PEGDA and 6% PEGMA.

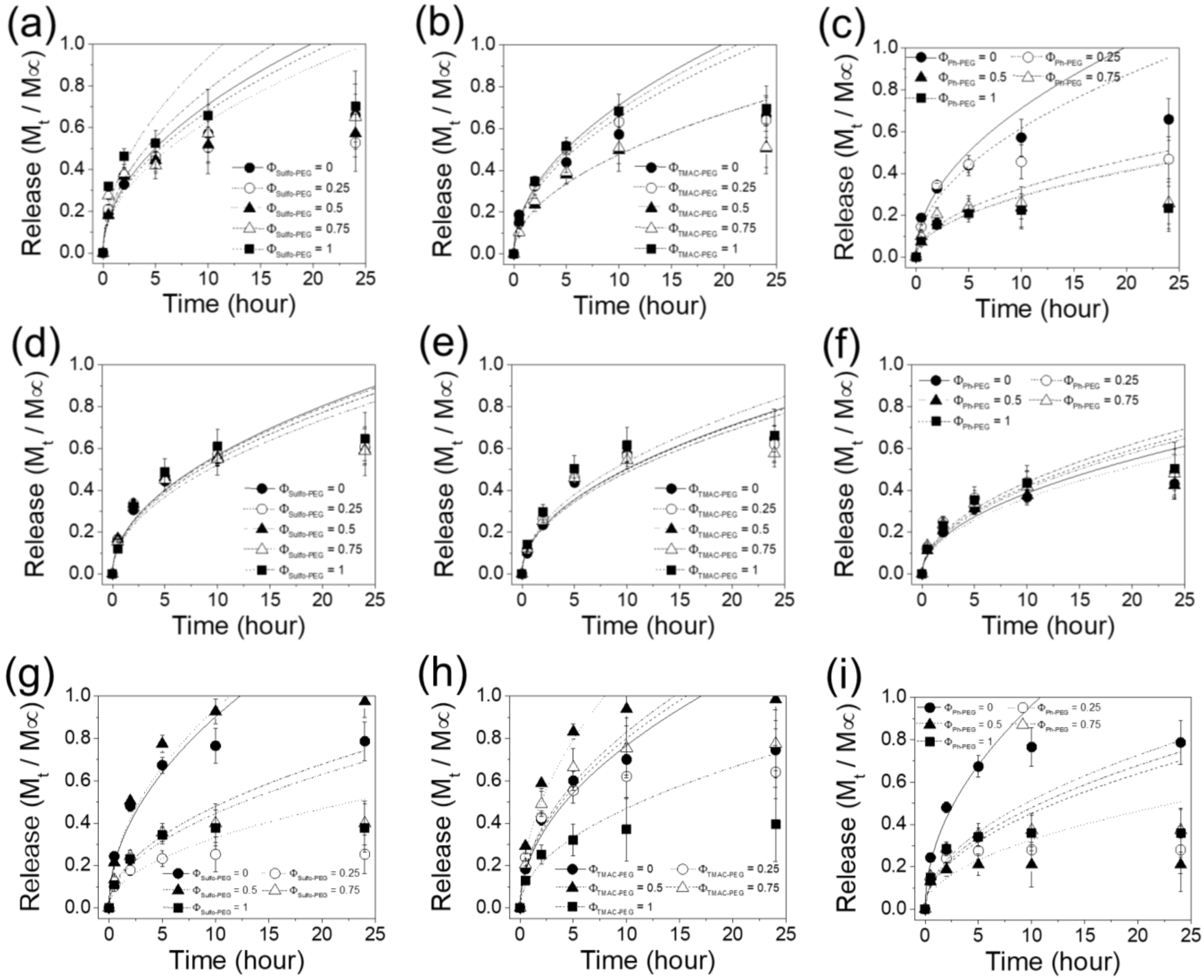

FIGURE S9. Release profiles of insulin from (a, d, g) Sulfo-PEG hydrogels, (b, e, h) TMAC-PEG hydrogels, and (c, f, i) Ph-PEG hydrogels. The profiles were fitted with Eq. (2). The PEGDA and PEGMA concentrations of the hydrogels were (a-c) 8 % and 4 %, (d-f) 10 % and 2 %, and (g-i) 6 % and 6 %.

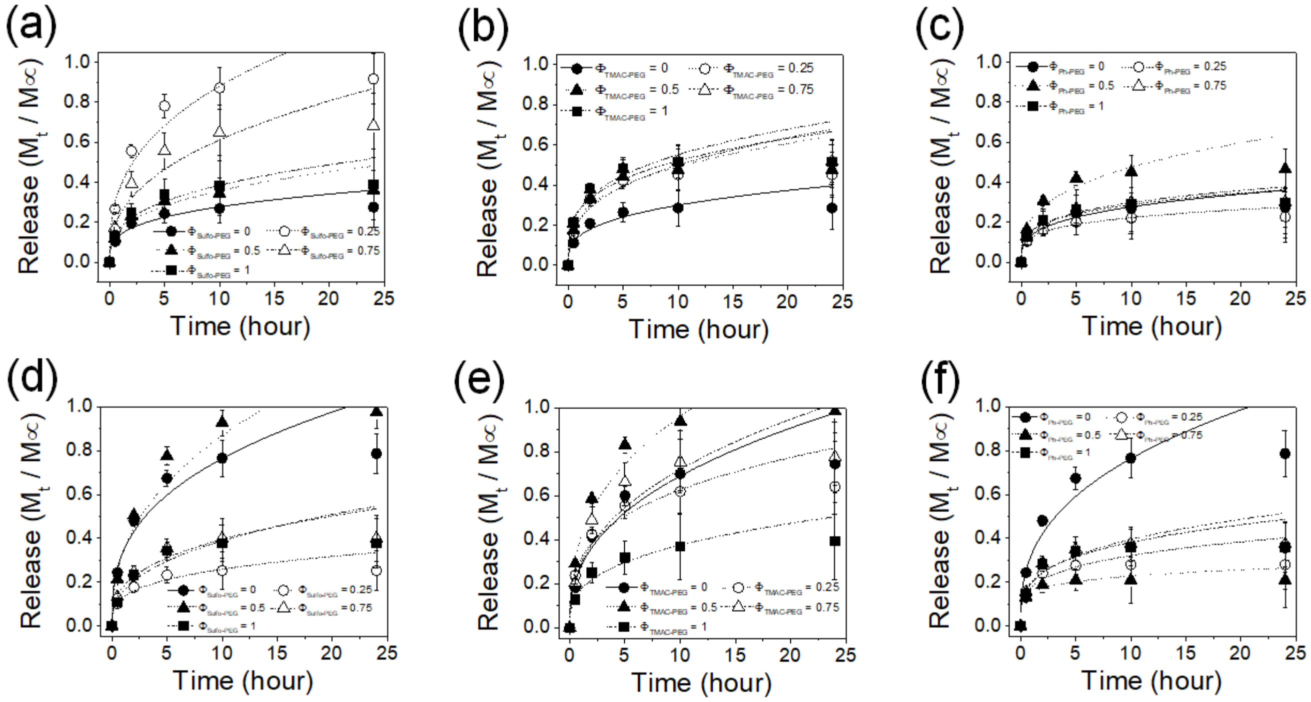

FIGURE S10. Release profiles of insulin from (a, d) Sulfo-PEG hydrogels, (b, e) TMAC-PEG hydrogels, and (c, f) Ph-PEG hydrogels. The profiles were fitted with Eq. (1). (a-c) 10% PEGDA and 2% PEGMA, (d-f) 6% PEGDA and 6% PEGMA.

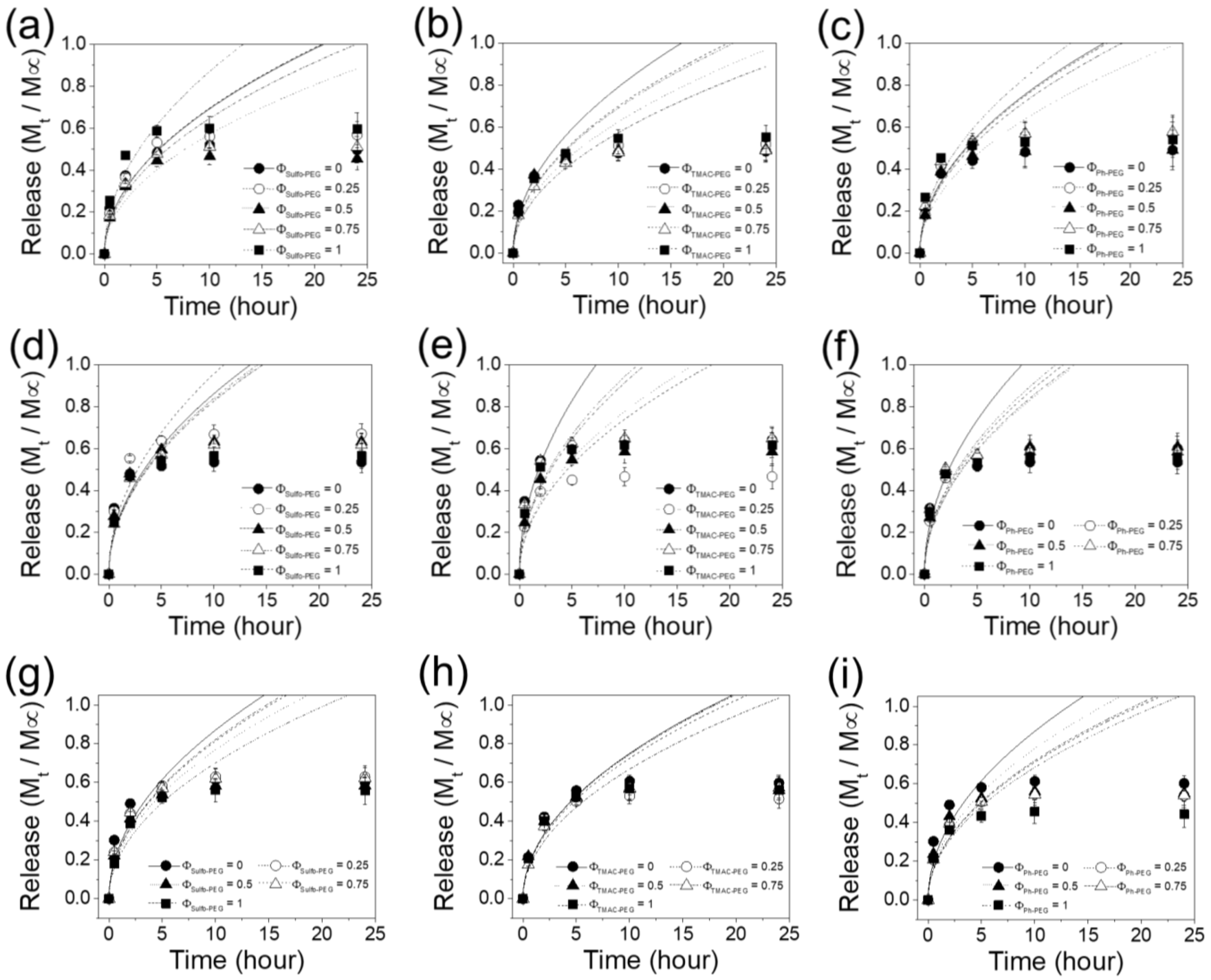

FIGURE S11. Release profiles of trypsin from (a, d, g) Sulfo-PEG hydrogels, (b, e, h) TMAC-PEG hydrogels, and (c, f, i) Ph-PEG hydrogels. The profiles were fitted with Eq. (2). The PEGDA and PEGMA concentrations of the hydrogels were (a-c) 8 % and 4 %, (d-f) 10 % and 2 %, and (g-i) 6 % and 6 %.

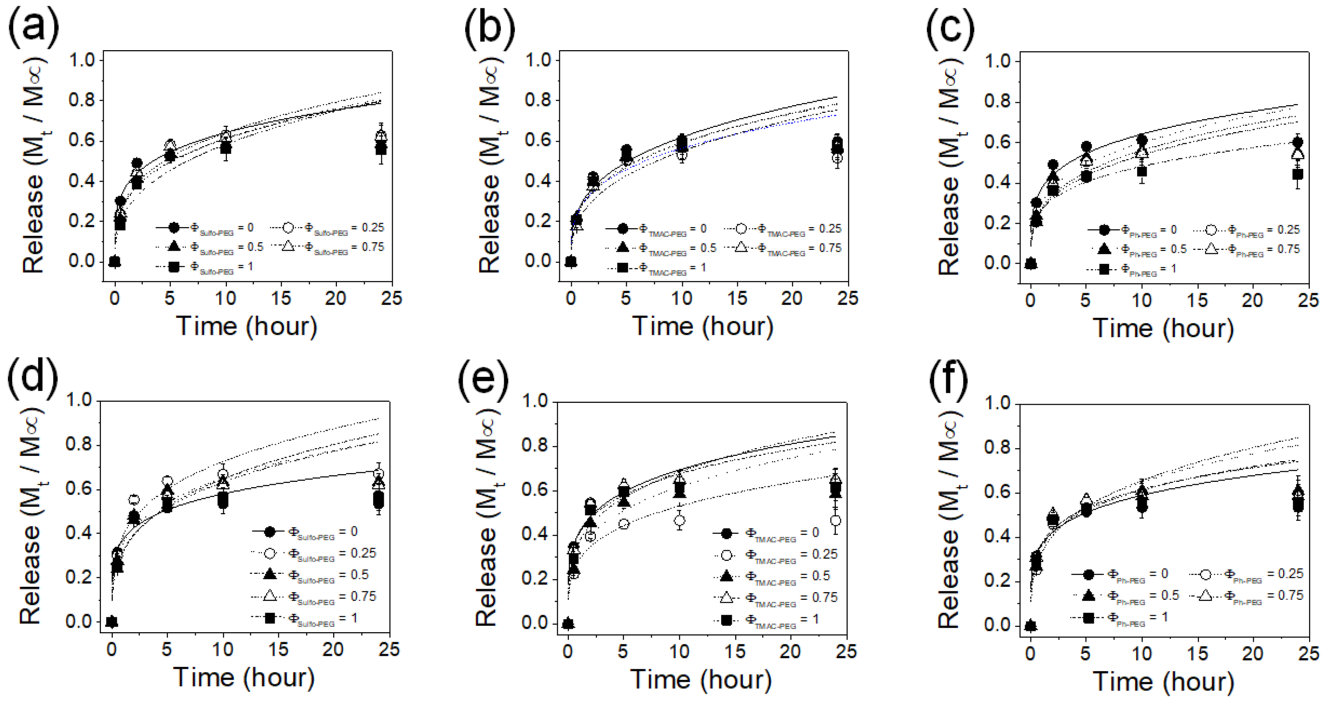

FIGURE S12. Release profiles of trypsin from (a, d) Sulfo-PEG hydrogels, (b, e) TMAC-PEG hydrogels, and (c, f) Ph-PEG hydrogels. (a-c) 10% PEGDA and 2% PEGMA, (d-f) 6% PEGDA and 6% PEGMA.

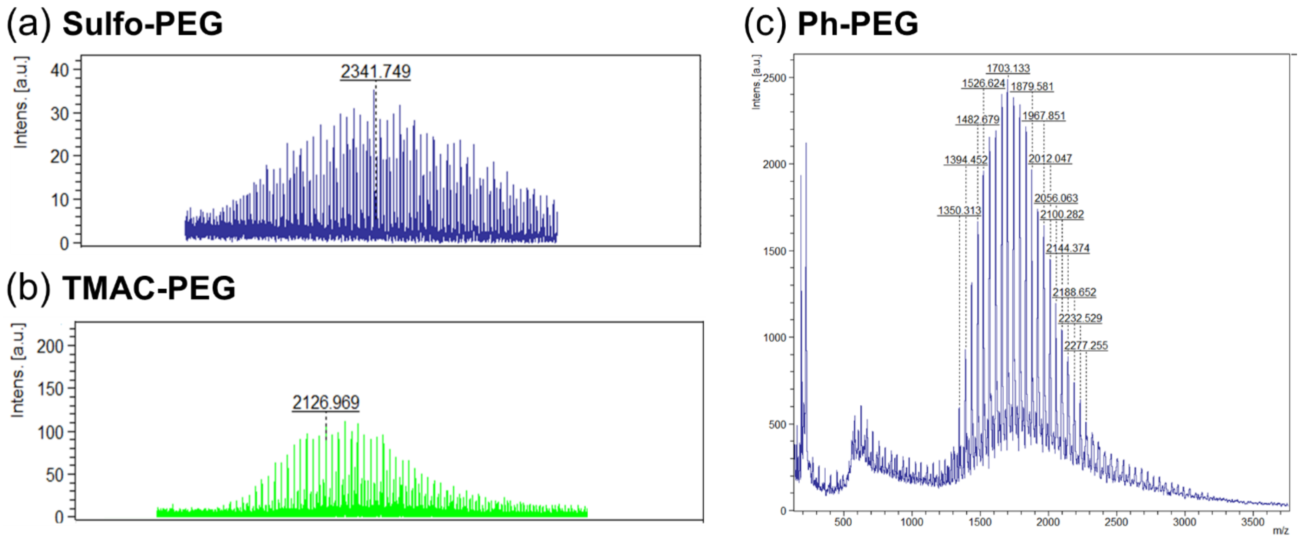

FIGURE S13. The molecular weight distributions of (a) Sulfo-PEG, (b) TMAC-PEG, and (c) Ph-PEG, determined from MALDI-TOF mass spectrometry.
